# Multi-polygenic scores in psychiatry: From disorder specific to transdiagnostic perspectives

**DOI:** 10.1002/ajmg.b.32951

**Published:** 2023-06-19

**Authors:** Yingjie Shi, Emma Sprooten, Peter Mulders, Janna Vrijsen, Janita Bralten, Ditte Demontis, Anders D. Børglum, G. Bragi Walters, Kari Stefansson, Philip van Eijndhoven, Indira Tendolkar, Barbara Franke, Nina Roth Mota

**Affiliations:** 1 Department of Human Genetics, Radboud University Medical Center, Nijmegen, The Netherlands; 2 Donders Institute for Brain, Cognition and Behaviour, Radboud University, Nijmegen, The Netherlands; 3 Department of Cognitive Neuroscience, Radboud University Medical Center, Nijmegen, The Netherlands; 4 Department of Psychiatry, Radboud University Medical Center, Nijmegen, The Netherlands; 5 Pro Persona Mental Health Care, Depression Expertise Centre, Nijmegen, The Netherlands; 6 Department of Biomedicine/Human Genetics, Aarhus University, Aarhus, Denmark; 7 The Lundbeck Foundation Initiative for Integrative Psychiatric Research, iPSYCH, Copenhagen, Denmark; 8 Center for Genomics and Personalized Medicine, Aarhus, Denmark; 9 deCODE Genetics, Reykjavík, Iceland; 10 Faculty of Medicine, University of Iceland, Reykjavík, Iceland

**Keywords:** factor analysis, polygenic risk scores, psychiatric disorders, transdiagnostic approach

## Abstract

The dense co-occurrence of psychiatric disorders questions the categorical classification tradition and motivates efforts to establish dimensional constructs with neurobiological foundations that transcend diagnostic boundaries. In this study, we examined the genetic liability for eight major psychiatric disorder phenotypes under both a disorder-specific and a transdiagnostic framework. The study sample (*n*= 513) was deeply phenotyped, consisting of 452 patients from tertiary care with mood disorders, anxiety disorders (ANX), attention-deficit/hyperactivity disorder (ADHD), autism spectrum disorders, and/or substance use disorders (SUD) and 61 unaffected comparison individuals. We computed subject-specific polygenic risk score (PRS) profiles and assessed their associations with psychiatric diagnoses, comorbidity status, as well as cross-disorder behavioral dimensions derived from a rich battery of psychopathology assessments. High PRSs for depression were unselectively associated with the diagnosis of SUD, ADHD, ANX, and mood disorders (*p* < 1e-4). In the dimensional approach, four distinct functional domains were uncovered, namely the negative valence, social, cognitive, and regulatory systems, closely matching the major functional domains proposed by the Research Domain Criteria (RDoC) framework. Critically, the genetic predisposition for depression was selectively reflected in the functional aspect of negative valence systems (*R*^2^= 0.041, *p*= 5e-4) but not others. This study adds evidence to the ongoing discussion about the misalignment between current psychiatric nosology and the underlying psychiatric genetic etiology and underscores the effectiveness of the dimensional approach in both the functional characterization of psychiatric patients and the delineation of the genetic liability for psychiatric disorders.

## 1 | INTRODUCTION

Psychiatric disorders are among the most common, disabling, and costly diseases in humans (GBD 2016 DALYs and HALE Collaborators, 2017), and yet, science falls short in understanding their etiopathogenesis. Conventional diagnostic frameworks, represented by the Diagnostic and Statistical Manual of Mental Disorders (DSM; American Psychiatric Association, 2013) and International Classification of Diseases (ICD; World Health Organization, 1993, p. 1), have often been employed as the scaffolding for mechanistic investigation and risk factor identification within case–control designs. However, limitations of adopting such discrete diagnostic frameworks in the research context have been well recognized, and distinct boundaries among diagnostic categories are challenged by the misalignment with patient profiles. Specifically, substantial differences in symptom profiles (Zimmerman et al., 2015; i.e., phenotypic heterogeneity) as well as neuronal features (Alnæs et al., 2019; Wolfers et al., 2018; i.e., biological heterogeneity) exist within the same diagnostic category, while patients with differently classified disorders could converge on overlapping symptomatology and/or pathological pathways (Craddock & Owen, 2010). The frequent observation of co-occurrence of multiple psychiatric disorders in clinical practice is closely tied with such heterogeneity and overlap. The high prevalence of psychiatric comorbidity (Plana-Ripoll et al., 2019) and the associated poorer clinical outcome require research to move beyond a single diagnosis and focus on the identification of transdiagnostic mechanisms. Several initiatives proposing dimensional alternatives have been established, such as the NIMH Research Domain Criteria (RDoC; Cuthbert & Insel, 2013) and the Hierarchical Taxonomy of Psychopathology (HiTOP; Kotov et al., 2017). In particular, the RDoC framework aims to explicate the neurobiological foundation of psychopathology using transdiagnostic biobehavioral domains, namely Negative Valence Systems, Positive Valence Systems, Cognitive Systems, Systems for Social Processes, Arousal/Regulatory Systems, and Sensorimotor Systems (Kozak & Cuthbert, 2016).

Recent psychiatric genetic studies have confirmed the overlapping genetic architecture among different disorders, pointing towards shared genetic substrates. The team efforts coordinating large-scale genome-wide association study (GWAS) meta-analyses have identified common genetic variations contributing to psychiatric disorders such as anxiety disorders (ANX; Purves et al., 2020), attention-deficit/hyperactivity disorder (ADHD; Demontis et al., 2023), autism spectrum disorder (ASD; Grove et al., 2019), bipolar disorder (BP; Mullins et al., 2021), schizophrenia (SCZ; Trubetskoy et al., 2022), and major depressive disorder (MDD; Wray et al., 2018). Building upon the GWAS knowledge base, genetic sharing among psychiatric disorders has been evaluated, which revealed substantial genetic overlap at the genomic level (The Brainstorm Consortium et al., 2018) from which over a hundred genetic variants exerting pleiotropic effects on more than one disorder could be identified (Grotzinger et al., 2022; Lee et al., 2019). The identification of the polygenic architecture and effect sizes carried by individual single nucleotide polymorphisms (SNPs) enables researchers to quantify the combined genetic susceptibility to disorders in the form of polygenic risk scores (PRSs), whose usefulness has been shown in risk prediction for common diseases (Khera et al., 2018) and treatment outcome prediction (Luykx et al., 2022), in identifying cross-disorder associations (Cross-Disorder Group of the Psychiatric Genomics Consortium, 2013), but also in investigating complex traits that are relevant to multiple disorders (Bralten et al., 2021). However, the relations of PRSs for different psychiatric disorders with transdiagnostic traits have rarely been investigated in clinically rather typical, highly comorbid cohorts.

In this study, we applied polygenic score analysis in a naturalistically recruited psychiatric cohort with high clinical complexity and comorbidity with two objectives. Under the conventional DSM-based framework, we aimed to assess the validity (i.e., significant association with target phenotype) and specificity (i.e., selective association with primary GWAS phenotype) of PRSs of major psychiatric disorders (multi-PRS) for different diagnostic outcomes. The PRSs were derived from the most recent and well-powered GWASs on ANX (Purves et al., 2020), ADHD (Grove et al., 2019), ASD (Grove et al., 2019), BP (Mullins et al., 2021), SCZ (Trubetskoy et al., 2022), MDD (Wray et al., 2018), depression (DEP; Howard et al., 2019), and cross-disorder diagnoses (cross-disorder; Lee et al., 2019) for patients from the recruited MIND-SET cohort and individuals free of any psychiatric disorder. Under a transdiagnostic dimensional framework, we explored the polygenic risk mapping to symptom and trait dimensions. For the latter, we first performed an exploratory factor analysis to explore latent structures in a broad range of psychopathological assessments of psychiatric, personality, and psychological symptoms and traits. Individuals’ representations on the derived functional dimensions were then examined with regard to the PRS and their comorbidity status.

## 2 | PATIENTS AND METHODS

### 2.1 | The MIND-SET cohort

The study sample MIND-SET cohort (Eijndhoven et al., 2022) was first established in Nijmegen, The Netherlands, in 2015. Recruited from the outpatient population of the Department of Psychiatry at Radboud University Medical Center, the sample is composed of adult (≥18 years) patients with a clinical diagnosis in at least one of five disorder categories (i.e., mood disorders, ANX, ADHD, ASD, and/or substance-related disorders). Individuals with current psychosis, IQ lower than 70, or inadequate command of the Dutch language were excluded from the study. A comparison group with similar demographics as the patients but free of any previous or current psychiatric disorders was recruited from the local population. Written informed consent was obtained from all participants included in the study. The study was approved by the local medical ethics committee (Commissie Mensgebonden Onderzoek Arnhem-Nijmegen).

### 2.2 | Disorder diagnosis

The diagnosis of patients was confirmed by a trained clinician during a structured interview. The absence of a lifetime psychiatric diagnosis in the control group was confirmed using the same diagnostic instruments via telephone interview. Mood disorders and ANX were diagnosed by means of the Structured Clinical Interview for DSM-IV Axis I Disorders (SCID-I; First et al., 1997). For ASD and ADHD, a diagnosis was provided based on the results from the Dutch Interview for the Diagnosis of ASD in adults (NIDA; Vuijk, 2016) and Diagnostic Interview for ADHD in adults (DIVA; Kooij & Francken, 2010), respectively. Substance use disorders (SUD) were diagnosed according to DSM-5 criteria and an adapted version of the Measurements in the Addictions for Triage and Evaluation (MATE; Schippers et al., 2010). Patients with SCZ and other psychotic disorders based on SCID-I were excluded. A detailed overview of the individual diagnoses included in each abovementioned disorder category and all participants’ demographic information is presented in Tables S1 and S2, accordingly. Individuals were identified as having comorbid disorders if they had diagnoses that fell into more than one of the disorder categories of mood disorders, ANX, ADHD, ASD, and SUD (i.e., only comorbidity between disorder categories was considered).

### 2.3 | Symptoms/trait questionnaires and exploratory factor analysis

A rich test battery was utilized to characterize the study sample with regard to disorder-related psychiatric symptoms, personality traits, and other psychological traits. Table S3 provides an overview of these questionnaires and their subscales used in the subsequent factor analysis. Further, the level of dysfunction in daily life of participants was assessed. The self-report World Health Organization Disability Assessment Schedule (WHODAS) 2.0 (Wenzel, 2017) was used to measure disability in six domains of functioning (i.e., cognition, mobility, self-care, getting along, life activities, and participation), and the Outcome questionnaire-45 (OQ-45; de Jong et al., 2007) was used to measure subjective experiences and social functioning in domains of symptom distress, interpersonal relations, and social role.

To derive transdiagnostic dimensions measured by the scales in Table S3, exploratory factor analysis (maximum likelihood estimation, oblique rotation) was performed based on 387 participants (327 patients) who had completed the entire test battery. The same analysis was previously conducted in a subset of participants from the same cohort, as described before (Mulders et al., 2022). A four-factor solution outperformed the simulated eigenvectors in the parallel analysis (Figure S1; Horn, 1965), which was in line with the scree-plot.

### 2.4 | Base GWAS datasets

We used the most recent and well-powered GWASs for both single psychiatric categories, including ANX (Purves et al., 2020), ADHD (Grove et al., 2019), ASD (Grove et al., 2019), BP (Mullins et al., 2021), SCZ (Trubetskoy et al., 2022), MDD (Wray et al., 2018), as well as broader disorder phenotypes, including DEP (Howard et al., 2019), and Cross-disorder (Lee et al., 2019) as the bases to derive PRSs for each participant of the MIND-SET cohort (see Table S4 for an overview of the datasets). The DEP GWAS included both cases who had clinically ascertained diagnosis of MDD (43 k, as described in Wray et al., 2018) and cases of “broad DEP” who reported help-seeking behavior for mental health difficulties (128 k, as described in Howard et al., 2018). All summary statistics from these datasets, except for ADHD, are publicly available. To our knowledge, none of the MIND-SET participants was included in any of the base GWAS samples. Any duplicated SNPs, ambiguous SNPs, multiallelic SNPs, and SNPs with minor allele frequency (MAF) lower than 0.01, or INFO score lower than 0.9 (if available) were removed from the PRS analysis.

### 2.5 | Genotyping, quality control, and imputation of the target dataset

The MIND-SET cohort was genotyped on the Infinium Global Screening Array (GSA-24 v3.0). The bioinformatics pipeline Ricopili (Lam et al., 2020; version from 2019_Oct_15.001), developed by the Psychiatric Genomics Consortium (PGC) Statistical Analysis Group, was employed to perform quality control and imputation on the genotyped data. To comply with the informed consent of the participants of the MIND-SET study, we removed variants known to be causative of diseases or disorders (i.e., pathogenic and likely pathogenic) in the genotyped data. We first excluded the variants within the pathogenic genes from the most recent list of ACMG (SF v2.0) genes recommended for return of secondary findings in clinical sequencing (Kalia et al., 2017). This step was conducted before performing any (pre-) processing on the genotyped sample in order to eliminate their impact on the imputation of other variants. MAF filter of 0.01 was applied after imputation to further remove the rare pathogenic variants that were imputed back to the data.

Several filters were applied to exclude individuals and SNPs of low genotyping quality: SNP call rate < 0.95 (prefilter), subject call rate < 0.98 for both cases and controls, autosomal heterozygosity deviation (*F*_HET_) outside ± 0.20, sex mismatch between genetic and phenotypic data, SNP call rate < 0.98, differences in SNP missingness between cases and controls > 0.02, SNP Hardy–Weinberg equilibrium *p* < 10^−6^, and invariant SNPs. To address population stratification, we performed principal component analysis (PCA) on the preprocessed data and removed the genetic outliers that were more than three standard deviations beyond the center of the European reference cluster in the 1000 Genome Project (Fairley et al., 2020). Further, overlapped/related individuals with pi-hat values > 0.2 were removed.

The imputation process was implemented by combining the Ricopili structure with the Michigan Imputation Server (Das et al., 2016; https://imputationserver.sph.umich.edu). After alignment with the reference panel, the genotypes from 22 autosomal chromosomes were phased (Eagle v2.4) and then imputed (Minimac 4) on the online server. We used the largest reference panel available, the Haplotype Reference Consortium (HRC) panel (r1.1 2016, Kretzschmar et al., 2016), which consists of 39 million SNPs from 32,470 samples of predominantly European ancestry. The imputed data was then integrated back to the Ricopili structure and best-guess genotypes were generated if the posterior probability of one of the genotypes was higher than 0.8 (otherwise it was assigned as missing). SNPs with missing rate higher than 0.02 were excluded from subsequent analysis, along with the ones with imputation quality (INFO score) lower than 0.9 and MAF lower than 0.01. PCA was performed again on the best guess genotypes (not including reference panels). Considering our sample size and the recommendation of RICOPILI pipeline (Lam et al., 2020), we included the first four derived principal components (PCs) as covariates in the subsequent polygenic score analyses, in addition to age and sex. As a sensitivity analysis, we also tested the models with levels of education as an additional covariate.

### 2.6 | PRSs calculation and association tests

For each individual in the MIND-SET cohort, PRSs for the eight GWAS bases mentioned in the above section were created using PRSice 2.3.3 (Choi & O’Reilly, 2019). Mismatching SNPs that could not be resolved by strand flipping were removed. Clumping was performed using a linkage disequilibrium *r*^2^ threshold of 0.1 and a sliding window of 250 kb to ensure independence among SNPs. A priori sets of 10 *p*-value thresholds (*p*_T_; i.e., 5e-8, 1e-6, 1e-4, 0.001, 0.01, 0.05, 0.1, 0.2, 0.5, and 1) were applied to the base GWASs to compute different genome-wide PRSs for each subject, and the best-fit PRS *p*_T_ (i.e. the most strongly associated PRS *p*_T_) for each outcome of interest (i.e., diagnostic outcomes, factor dimensions) was identified and retained. To avoid overfitting during the optimization of *p*_T_, we computed empirical *p*-values (*p*_emp_) by performing 10,000 permutations using random phenotypes to generate the null *p*-value distribution (Choi & O’Reilly, 2019). Additionally, we adopted a stringent Bonferroni-corrected threshold *α*= 0.001 to account for the multiple tests (~50 tests) performed with different base disorders/traits and the outcome disorder statuses. We provided a rough estimation of the statistical power of PRSs for their corresponding phenotype in Table S5 using R package “avengeme” (Dudbridge, 2013). Pairwise correlations among different disorder PRSs were shown in Figure S2. To further validate the results, we also implemented a Bayesian-based continuous shrinkage (PRS-CS) method (Ge et al., 2019) to assess the consistency of different PRS-scoring methods.

PRSs were used as predictors in both simple and multiple logistic regression models for diagnostic outcomes, and simple linear regression for factors scores. Variance inflation factors were calculated to detect potential multicollinearity in the multiple regression models. The proportion of variance explained by the PRSs in all outcomes was estimated by Nagelkerke’s pseudo-*R*^2^, computed as the difference between the *R*^2^ of the full model, containing the PRS and the covariates (i.e., age, sex, and the first four PCs), and the *R*^2^ of the null model, containing only the covariates. One-way ANOVAs were applied to test the differences in PRSs and factor loadings among groups with different comorbidity statuses.

## 3 | RESULTS

### 3.1 | Diagnoses and comorbidities in the MIND-SET cohort

**FIGURE 1:**
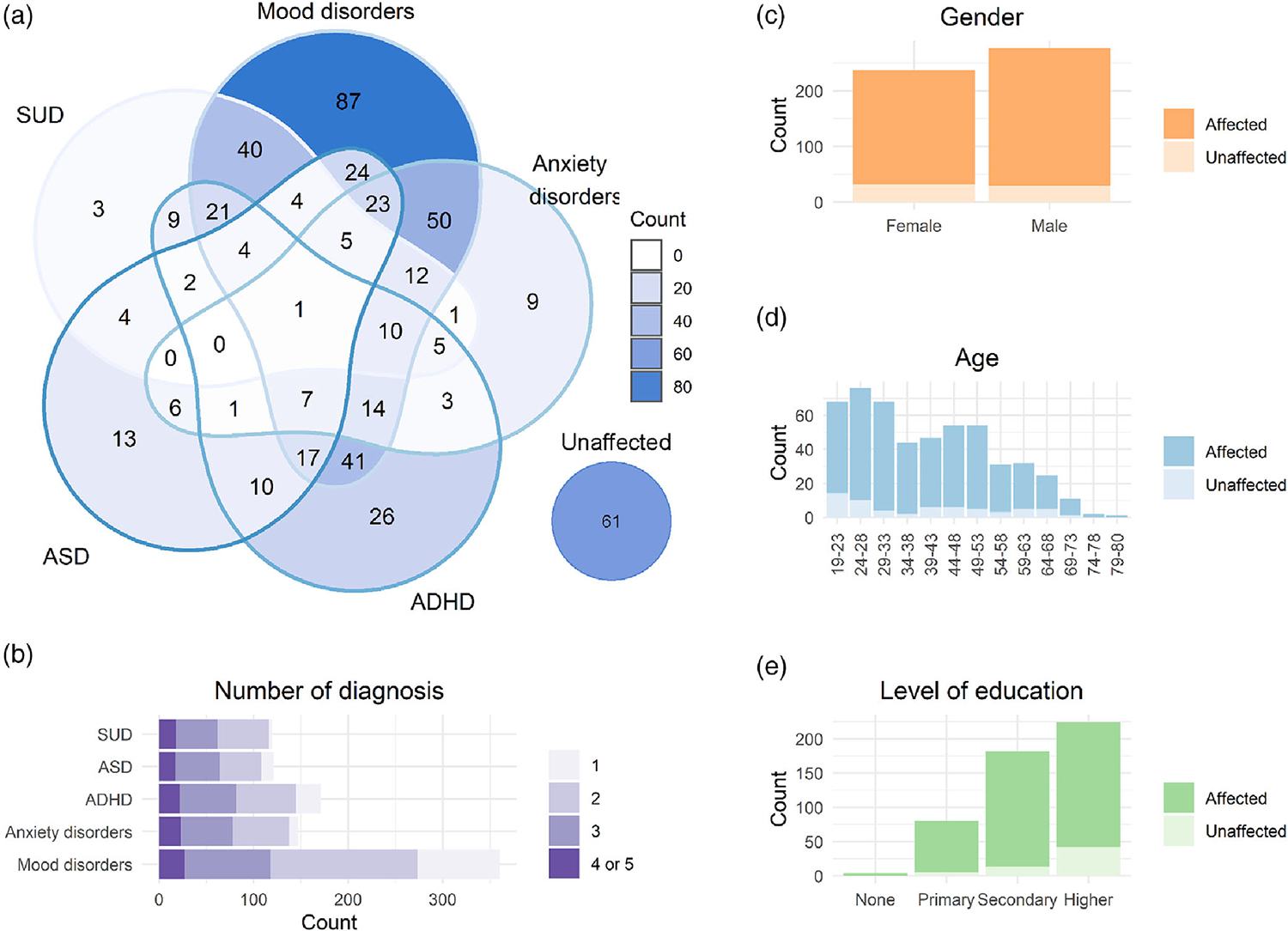
MIND-SET participant characteristics, psychiatric diagnoses, and comorbidity status. Venn diagram of the five disorder groups (a) and the summary of the number of diagnosis patients had (b). Multiple diagnoses within the same disorder group were regarded as one. The distribution of gender (c), age (d), and level of education (e) of cases and the unaffected comparison group. ASD, autism spectrum disorders; ADHD, attention-deficit/hyperactivity disorder; SUD, substance use disorders. [Color figure can be viewed at wileyonlinelibrary.com]

A total of 513 individuals of European ancestry were included in the study: 452 had at least one diagnosis of mood disorder, ANX, ADHD, ASD, and/or SUD, and 61 were sex- and age-matched unaffected individuals. An overview of the refined diagnoses within each disorder category is presented in Table S1. Among the patients, 80% (*n* = 360) had at least one diagnosis in the mood disorders spectrum, 33% (*n* = 147) had at least one ANX, 38% (*n* = 171) had ADHD, 27% (*n* = 121) had ASD, and 27% (*n* = 121) had SUD. Psychiatric comorbidity was highly prevalent in the MIND-SET cohort: 70% of the patients fell into at least two diagnostic categories, and 28% into three or more. As shown in Figure 1a, mood disorders in combination with ANX, ADHD, or SUD were among the most common comorbidities in the current cohort.

## 4 | MULTI-PRS AND DISORDER DIAGNOSTIC STATUS

**FIGURE 2:**
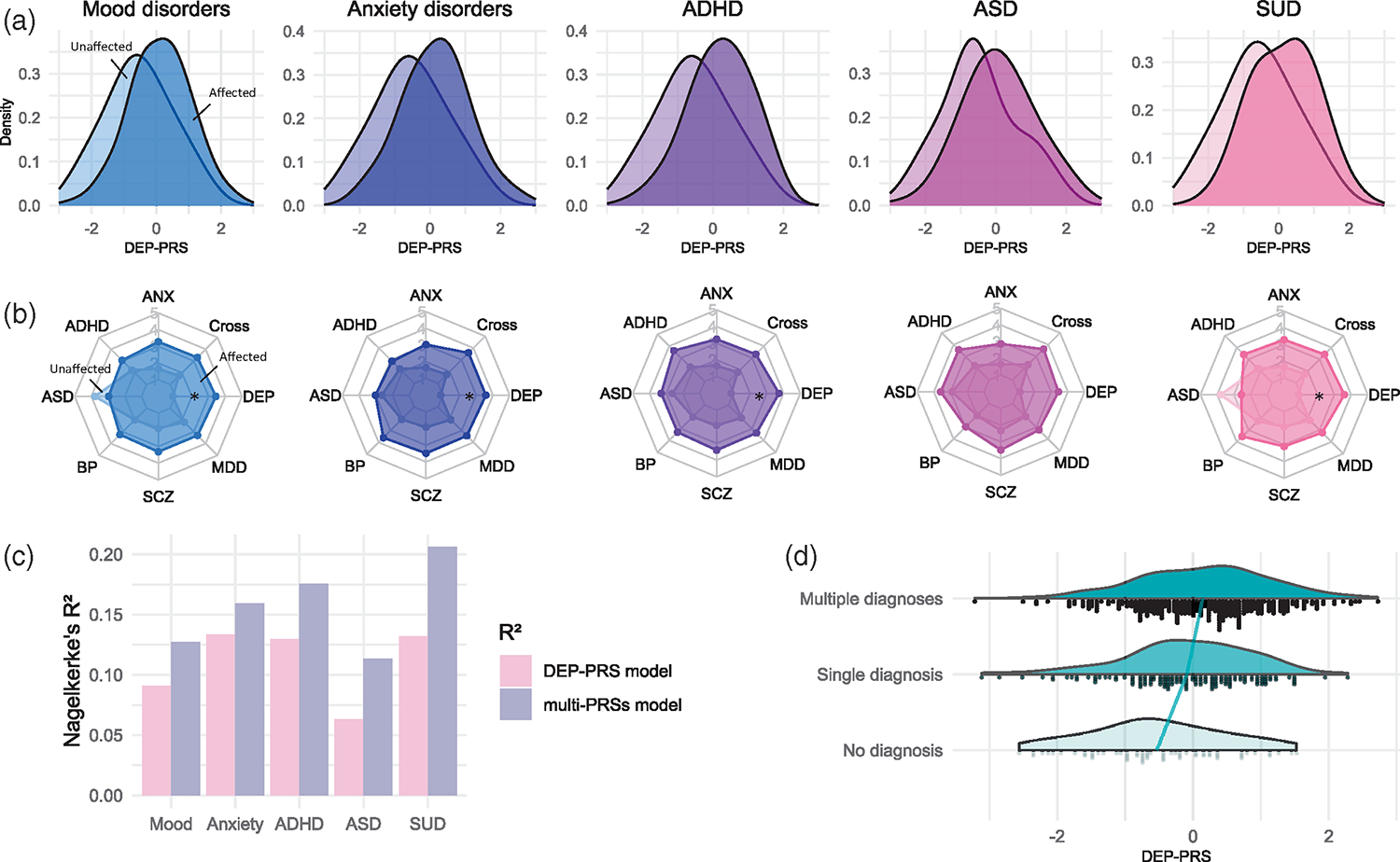
Multipolygenic risk score (PRS) profile for different disorder categories and comorbidities. (a) PRS distributions for broad depression (DEP-PRS) of unaffected (light color) and patient samples (dark color) of the disorder shown under each subplot. See Figure S3 for distributions of anxiety disorders (ANX)-, attention-deficit/hyperactivity disorder (ADHD)-, autism spectrum disorder (ASD)-, bipolar disorder (BP)-, schizophrenia (SCZ)-, major depressive disorder (MDD)-, and cross-disorder-PRS. (b) Eight-axis PRS profiles for each disorder group, with each axis representing the PRS based on one genome-wide association study. PRSs were constructed using the *p*_T_ that yielded the strongest associations with the outcome of interest. *p_emp_ <0.001. (c) Variance explained by DEP-PRS as the single predictor in comparison to eight PRS predictors. *R*^2^ for the null model (age, sex, and four PCs) has been subtracted from both model. (d) Group differences in PRSs for depression with regard to individuals’ number of diagnoses (group with no diagnosis *N* = 61, single diagnosis *N* = 138, and multiple diagnoses *N* = 314). The reference line connects the average value for each group. [Color figure can be viewed at wileyonlinelibrary.com]

PRSs computed based on the broadly defined phenotype of DEP (i.e., DEP-PRS) were significantly associated not only with mood disorder status, but—even to a larger extent—with SUD, ADHD, and ANX (Tables 1 and S6, all at *p*_T_= 0.01). Figure S3 shows model fit for all tested thresholds. The results remain consistent using scores derived from the PRS-CS approach (Table S7), and also after adding levels of education as an additional covariate (Table S9). In contrast, neither single disorder-based PRSs (incl. ANX, ADHD, ASD, BP, SCZ, and MDD) nor the cross-disorder PRSs significantly explained the diagnostic status of any disorder category after Bonferroni correction. The PRS distributions of individuals within each disorder category and unaffected comparisons are depicted in Figure 2a for DEP and in Figure S4 for other PRSs. To assess the impact of the discovery sample size, we used a subset of broadly defined DEP GWAS sample (Wray et al., 2018; *n* = 323 k) to construct subset-DEP-PRS, which still showed the strongest associations with several disorder diagnoses despite being the third largest discovery sample (Table S6).

Combining the genetic risks across different base disorders, we present in Figure 2b the multiaxis genetic risk profiles for the affected and unaffected groups. Since group differences in several disorder categories were found also for the ADHD-, ANX-, and SCZ-PRSs at an uncorrected significance threshold (Table 1), we tested whether adding these PRSs to the model would improve prediction for disorder outcome compared with the DEP-PRS alone. Using all eight PRSs as predictors, the multiple regression model explained 3.07%–7.47% more variance than the model with DEP-PRS as the single predictor (Figure 2c), but did not yield statistically significant improvement on the model fit for the disorder outcomes (Table S10).

To further test whether DEP-PRS was related to disorder comorbidity status, we compared the DEP-PRS among groups of unaffected individuals, patients with a single disorder and the comorbid group (Figure 2d). The results suggested a significant overall DEP-PRS effect on comorbidity status (*F* = 12.960, *p* = 3e-6): patients with comorbid conditions had nominally higher DEP-PRS compared with patients with only one diagnosis (*t* = 2.335, *p* = 0.050), who had nominally higher DEP-PRS than unaffected individuals (*t* = 2.936, *p* = 0.009).

## 5 | DATA-DRIVEN FUNCTIONAL DIMENSIONS

**FIGURE 3:**
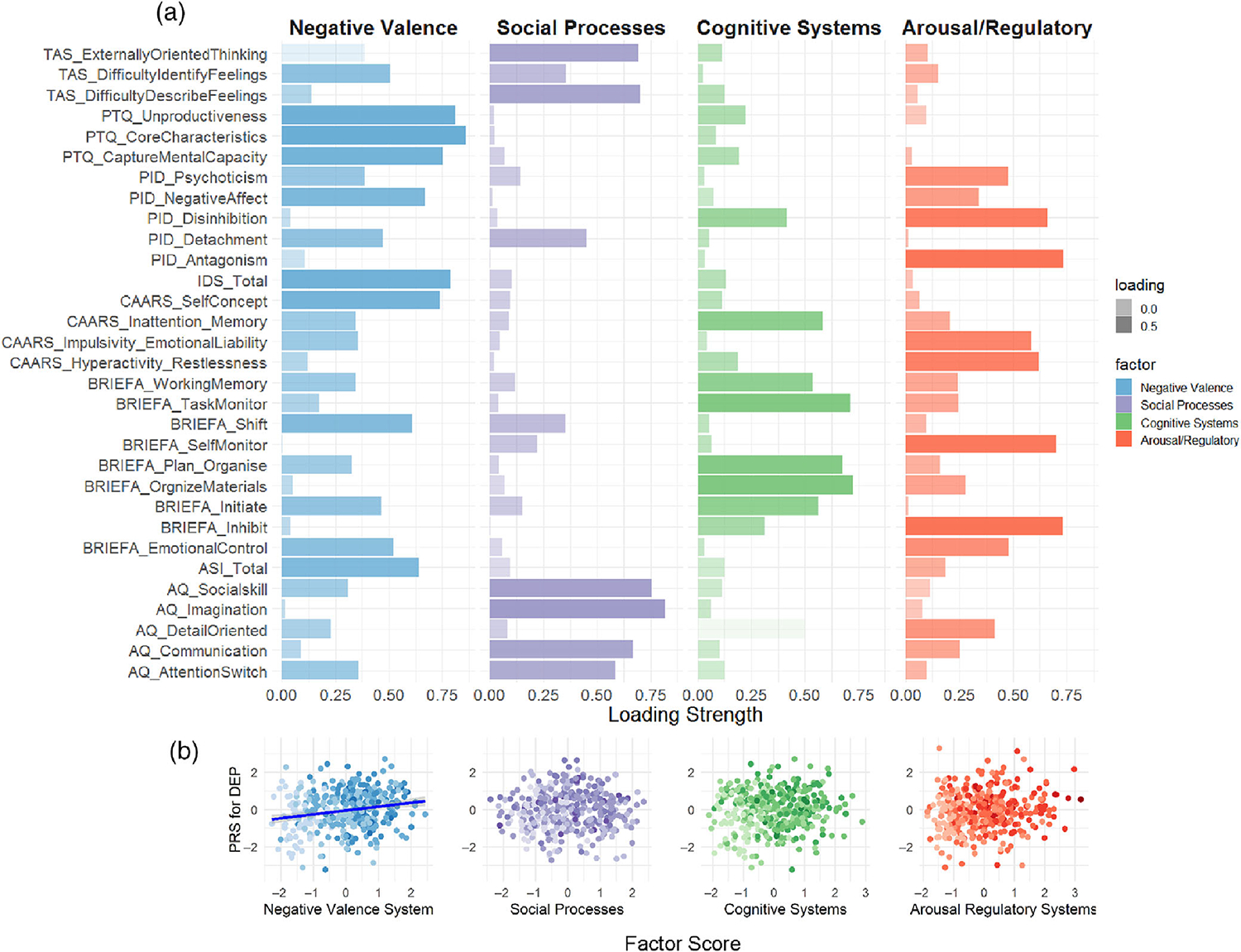
Rotated component matrix of four factors resulting from factor analysis of psychopathology measurements. (a) Four factors were retained after parallel analysis and were interpreted in the column headers. The *Y*-axis shows the (sub)scales included in the analysis following the naming scheme—“questionnaire name abbreviation_subscale.” Please consult Table S3 for a detailed list of the questionnaires included in the exploratory factor analysis. The component matrix contains the factor loadings (Pearson correlations between items and components) on each subscale with color intensity corresponding to the loading strength. (b) Individuals’ polygenic risk score (PRS) for depression in relation to their scoring on each factor dimension. Line of best fit is plotted for the negative valence factor, which is significantly (*p* < 0.001) correlated with PRS for depression. Color intensity is scaled according to the number of diagnoses. [Color figure can be viewed at wileyonlinelibrary.com]

**FIGURE 4:**
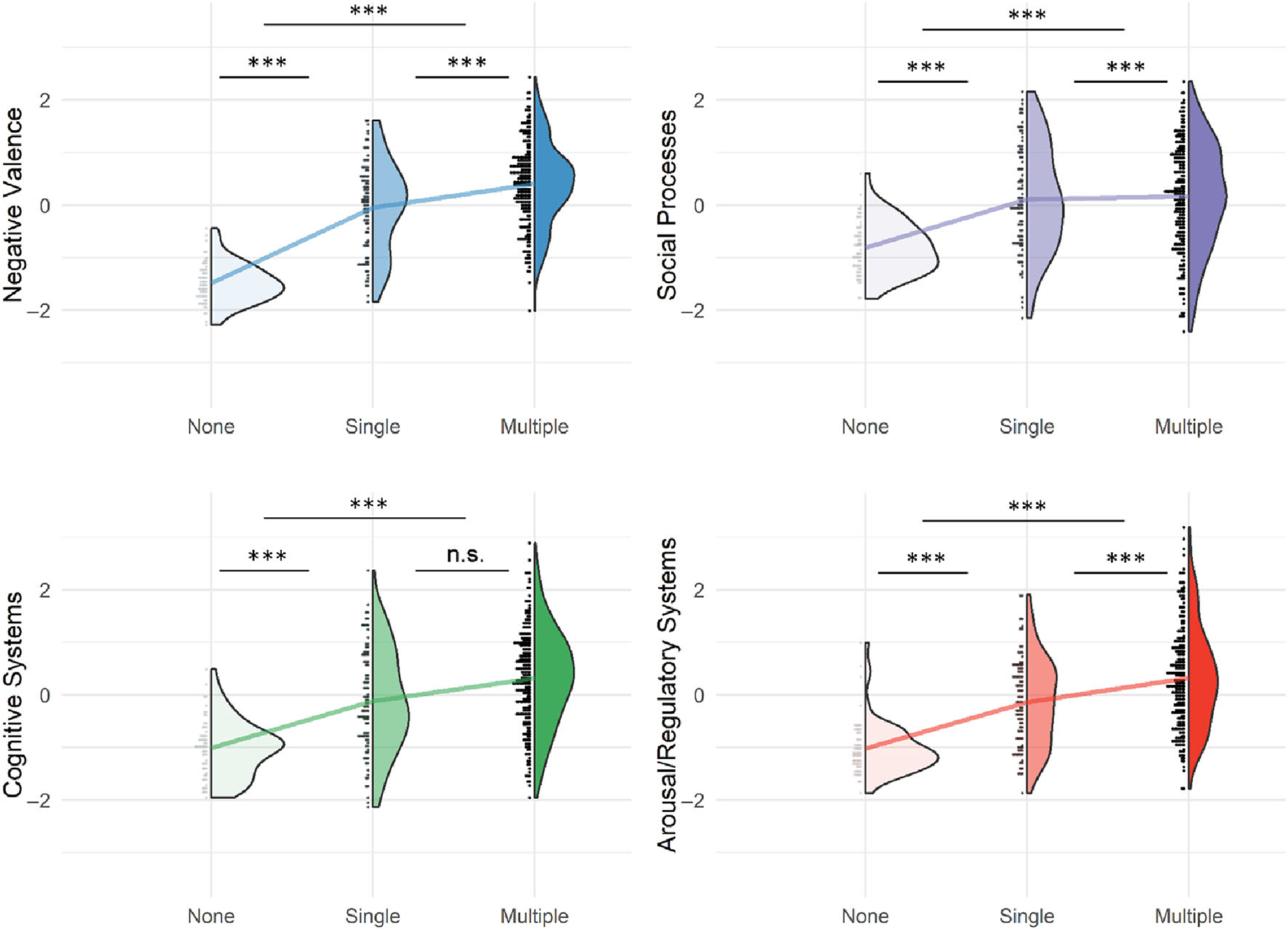
Group differences in factor loading with regard to comorbidity status. Individuals’ loadings for four-factor dimensions were stratified by whether the individual had no disorder diagnosis (*N* = 60), single diagnosis (*N* = 95), or more than one diagnosis (multiple; *N* = 232). The line in each subplot connects the mean of the three groups. Higher score indicates higher dysfunction. The post hoc tests were performed using Tukey method. *** *p* < 0.001. n.s., not significant. [Color figure can be viewed at wileyonlinelibrary.com]

Converging a wide array of psychopathology assessments into cross-disorder constructs, the factor analysis of 31 (sub)scales of psychiatric, personality, and psychological traits measured in MIND-SET (Table S3) yielded four factors, which together explained 67.3% of the variance (KMO = 0.948, Bartlett’s test *p* < 0.001). These four factors matched the previous finding using a subsample of the same cohort with a highly similar component matrix (Mulders et al., 2022), in which the interpretation of the factors roughly corresponded to previously defined RDoC domains (Cuthbert & Insel, 2013; Kozak & Cuthbert, 2016; Figure 3a): the first factor related to negative thinking, emotions, and poor self-concept across instruments (i.e., RDoC negative valence systems); the second factor summarized difficulties in social functioning (i.e., RDoC social processes); the third factor described cognitive abilities (i.e., RDoC cognitive systems); the last factor related to the ability in regulation and inhibition (i.e., RDoC arousal/regulatory systems). To evaluate the relevance of the derived factors to individuals’ functioning and disabilities, we assessed their relationship with self-rated quality of life measured using OQ-45 and WHODAS 2.0 scales. We found that all four factors had significant positive regression weights for the outcome of individuals’ subjective distress and social dysfunction measured in OQ-45, and the first three factors had significant positive regression weights for the outcome of overall disability measured with WHODAS 2.0 (Table S11); together, they explained the outcomes with an adjusted *R*^2^ of 0.81 and 0.65, respectively. Different disorder categories were represented by distinct factor profiles, which resembled their clinical presentations (Figure S5). For example, patients with ADHD loaded highly on the dysfunction in cognitive and arousal/regulatory systems, whereas patients with ASD had higher dysfunction loading in the social processes. Compared with unaffected individuals, all patients scored higher on the loading of dysfunction on all factors (Figure 4 and Table S12). Compared with the group with only one diagnosis, the comorbid group had significantly higher loadings for dysfunction for negative valence systems (*t* = 4.928, *p* = 3e-6), social processes (*t* = 4.025, *p* = 2e-4), and arousal/regulatory systems (*t* = 4.321, *p* = 5e-5). For cognitive systems, there was no significant difference in loading between groups with single and multiple diagnoses (*t* = 0.483, *p* = 0.878).

Since DEP-PRS was found to be significantly associated with multiple diagnostic status extending beyond mood disorders, we set out to test whether this PRS was associated with specific aspect(s) of behavioral functioning. PRSs derived from other disorders/phenotypes were not included in this analysis. We found that the DEP-PRS was significantly associated with the negative valence dimension (Figure 3b; *R*^2^ = 0.041, *p*_emp_ = 5e-4 at *p*_T_ = 0.01), but not with social processes (*R*^2^= 0.029, *p*_emp_ = 0.004 at *p*_T_ = 0.2), cognitive systems (*R*^2^= 0.001, *p*_emp_ = 0.960 at *p*_T_ = 0.01), or arousal and regulatory systems (*R*^2^ = 0.018, *p*_emp_ = 0.040 at *p*_T_ = 0.001). The results were consistent after adding levels of education as an additional covariate (Table S9).

## 6 | DISCUSSION

Bringing genetic metrics derived from case–control samples into a highly comorbid clinical cohort, our study provided a real-world assessment of the validity and specificity of psychiatric PRSs, with regard to both disorder-specific and transdiagnostic outcomes. Multi-PRS analysis revealed that the DEP-PRS outperformed all other PRSs and were significantly associated with the diagnostic statuses of SUD, ADHD, ANX, and mood disorders. We reproduced four transdiagnostic dimensions derived from a diversity of psychology and psychopathology measurements and revealed that one specific dimension—the negative valence system—was selectively associated with DEP-PRS.

Our association analyses with DSM diagnoses showed that the genetic propensity for a broadly defined depression phenotype (i.e., DEP, SNP heritability = 0.089) was significantly associated with disorder statuses outside of the mood disorders spectrum. With more than 75% of cases identified based on “minimal phenotyping” (i.e., a positive answer to the question “Have you seen a GP/psychiatrist for nerves, anxiety, stress or depression?”), the original GWAS on which the DEP-PRS was computed (excluding 23andMe cohort; Howard et al., 2019) is phenotypically much broader than GWAS using clinically ascertained (major) depression (Purves et al., 2020) and statistically more powerful given its sample size. Previous studies have addressed the fact that such broad phenotyping approaches might identify a genetic architecture that was not specific to the clinical form of MDD (Cai et al., 2020), but noticed that those can be highly useful for risk prediction and risk factor identification—especially given the convenience to reach large sample sizes (Mitchell et al., 2021).

Consistent with the previous factor analysis results from a smaller overlapping sample (Mulders et al., 2022), we observed four transdiagnostic domains (i.e., negative valence, social, and arousal/regulatory systems) among a broad battery of psychiatric, personality, and psychological assessments, which together explained up to 81% of individuals’ subjective experience of their functioning and disability. Importantly, the finding that DEP-PRS was selectively linked to the functioning of the negative valence system points to the direction of a shared domain of functioning that underlies its associations with multiple disorders. This underscores the importance of a paradigm shift in mechanistic investigations towards data-driven dimensional constructs that acknowledge the intertwined nature of different categorized psychiatric disorders. Uncovering the latent dimensions of psychopathology from dense symptom- and trait-level data, on the one hand, will help to identify individuals’ unique (dys-)functional profile and enable targeted interventions for specific functional spectra; on the other hand, it can provide neurobiological studies with an improved scaffolding to investigate underlying pathogenic processes. Although attempts of genomic enquiry on dimensional traits established in a data-driven fashion are still scarce, efforts of GWAS on theory-based psychopathology traits (e.g., extracted from clinical notes [McCoy et al., 2018], neurocognitive tests [de la Fuente et al., 2021], self-report assessments [Genetics of Personality Consortium, 2015]) are blooming and provide important leads for follow-up causal assessments.

With its broad relevance, the DEP-PRS explained different diagnostic outcomes in our cohort to a higher magnitude than previously reported in other cohorts (Howard et al., 2019; Kember et al., 2021). While an independent sample is required to further validate the predictive accuracy, we postulate that the contrast between highly severe and comorbid cases recruited from a specialized academic hospital and a “clean” comparison group free of any psychiatric history could have amplified the variance explained by the PRS. This would lead to a larger *R*^2^ compared with previous reports where patients may have had less complex, less comorbid clinical profiles, and controls were not always screened for other major psychiatric disorders (Howard et al., 2019). Furthermore, we recognize that the composition of disorders of the current cohort might align well with the constellation of psychiatric characteristics probed by such “minimal phenotyping” (Cai et al., 2020) definition of the broad DEP PRS, which will also give rise to a more effective PRS.

In the current study sample, the psychiatric heterogeneity spanned a large spectrum of mental health and functioning, including individuals free of mental health complaints and tertiary care patients with multiple diagnoses. Unlike previous studies where comorbid conditions were either ignored (i.e., participants are unscreened for other disorders) or treated as confounding variables or exclusion criteria, we addressed the topic of psychiatric comorbidity explicitly and characterized it on both the genetic and cross-disorder behavioral scales. We showed that individuals displaying comorbidity were bearing higher genetic liability and displayed a higher degree of dysfunction in most functional aspects. This adds functional and biological evidence to the large body of comorbidity literature showing phenotypic associations between psychiatric comorbidity and higher severity and more chronicity of impairment (e.g., Klein Hofmeijer-Sevink et al., 2012; Overbeek et al., 2002). Rather than regarding psychiatric disorders as distinct entities that deserve separate treatments on top of each other, it is crucial to acknowledge the shared underlying vulnerability factors and etiopathogenesis that push individuals to the higher end of the psychopathology spectrum.

The present study provides a thorough assessment of the validity and specificity of PRSs for major psychiatric disorders against categorical as well as dimensional outcomes by exploiting a clinically well-assessed cohort spanning a wide spectrum of psychopathology. However, several limitations need to be taken into account when interpreting the results. First, the low sample size of our cohort, especially that of the unaffected comparison group, could limit the statistical power and contribute to the lack of associations of PRSs other than DEP-PRS. Second, while a permutation procedure was performed to adjust association *p*-values, the observed phenotypic variance explained (*R*^2^) requires an independent sample to further evaluate the predictive accuracy of the PRSs. Third, only comorbidities among the defined nonpsychotic disorder categories (as opposed to within disorder categories) were considered, which may yield an incomplete picture of the functional and genetic characterization of psychiatric comorbidity.

In conclusion, our polygenic scoring analysis revealed low specificity to psychiatric disorders as defined by conventional classification systems, but enhanced specificity to data-driven functional domains. Domain-based genetic analyses targeting traits and symptoms not restricted to a single disorder or clinically ascertained group could help reduce the clinical and biological heterogeneity of the study sample and enable more fine-grained mapping to the biological basis of psychopathology at different levels. It also supports further initiatives of targeted treatments based on neurocognitive domains that eventually can provide an important avenue for psychiatric interventions.

## Supplementary Material

Supplementary materials

## Figures and Tables

**TABLE 1:** Variance explained (pseudo-R2 (pemp values)) in diagnosis status by PRSs for different psychiatric disorders.

Phenotype	ANX-PRS	ADHD-PRS	ASD-PRS	BP-PRS	SCZ-PRS	MDD-PRS	DEP-PRS	Cross-PRS
MoodDis	*0.035* (*0.027*)	0.013 (0.285)	0.010 (0.525)	0.020 (0.117)	*0.026* (*0.045*)	0.021 (0.121)	**0.091 (2e-4)**	*0.032* (*0.032*)
AnxDis	0.039 (0.104)	0.016 (0.438)	0.003 (0.981)	0.041 (0.062)	*0.043* (*0.043*)	0.038 (0.096)	**0.133 (5e-4)**	*0.052* (*0.036*)
ADHD	*0.048* (*0.042*)	*0.047* (*0.034*)	0.008 (0.804)	0.025 (0.181)	0.034 (0.070)	0.025 (0.195)	**0.130 (1e-4)**	0.034 (0.102)
ASD	0.039 (0.115)	0.033 (0.157)	0.006 (0.920)	0.019 (0.363)	0.029 (0.122)	0.023 (0.305)	*0.063* (*0.021*)	0.030 (0.173)
SUD	*0.060* (*0.030*)	0.030 (0.185)	0.022 (0.412)	0.033 (0.139)	0.024 (0.195)	0.025 (0.283)	**0.132 (2e-4)**	0.040 (0.103)

*Note:* The proportion of variance explained by each PRS in each of the five psychiatric disorder diagnoses was estimated by Nagelkerke’s pseudo-*R*^2^, computed as the difference between the *R*^2^ of the single PRS model, containing one PRS and the covariates (i.e., age, sex, and four PCs), and the *R*^2^ of the null model, containing only the covariates. Results from an alternative polygenic scoring approach (i.e., PRS-CS) are presented in Table S7. Associations that exceeded Bonferroni-corrected threshold of *p* = 0.001 were labeled in bold, and those exceeding the uncorrected threshold of *p* = 0.05 were labeled in italic.

Abbreviations: ANX, anxiety disorders; ADHD, attention-deficit/hyperactivity disorder; ASD, autism spectrum disorders; BP, bipolar disorder; SCZ, schizophrenia; MDD, major depressive disorder; DEP, depression; SUD, substance use disorders.

## Data Availability

The data that support the findings of this study are available from MIND-Set study. Restrictions apply to the availability of these data, which were used under license for this study. Data are available from the author(s) with the permission of MIND-Set study.

## References

[R1] Alnæs D, Kaufmann T, van der Meer D, Córdova-Palomera A, Rokicki J, Moberget T, Bettella F, Agartz I, Barch DM, Bertolino A, Brandt CL, Cervenka S, Djurovic S, Doan NT, Eisenacher S, Fatouros-Bergman H, Flyckt L, Di Giorgio A, Haatveit B, … Westlye LT (2019). Brain heterogeneity in schizophrenia and its association with polygenic risk. JAMA Psychiatry, 76(7), 739–748. 10.1001/jamapsychiatry.2019.0257 30969333 PMC6583664

[R2] American Psychiatric Association. (2013). Diagnostic and statistical manual of mental disorders. American Psychiatric Publishing.

[R3] Bralten J, Mota NR, Klemann CJHM, De Witte W, Laing E, Collier DA, de Kluiver H, Bauduin SEEC, Arango C, Ayuso-Mateos JL, Fabbri C, Kas MJ, van der Wee N, Penninx BWJH, Serretti A, Franke B, & Poelmans G (2021). Genetic underpinnings of sociability in the general population. Neuropsychopharmacology, 46(9), 1627–1634. 10.1038/s41386-021-01044-z 34054130 PMC8280100

[R4] Cai N, Revez JA, Adams MJ, Andlauer TFM, Breen G, Byrne EM, Clarke T-K, Forstner AJ, Grabe HJ, Hamilton SP, Levinson DF, Lewis CM, Lewis G, Martin NG, Milaneschi Y, Mors O, Müller-Myhsok B, Penninx BWJH, Perlis RH, … Flint J (2020). Minimal phenotyping yields genome-wide association signals of low specificity for major depression. Nature Genetics, 52(4), 437–447. 10.1038/s41588-020-0594-5 32231276 PMC7906795

[R5] Choi SW, & O’Reilly PF (2019). PRSice-2: Polygenic risk score software for biobank-scale data. GigaScience, 8(7), giz082. 10.1093/gigascience/giz082 31307061 PMC6629542

[R6] Craddock N, & Owen MJ (2010). The Kraepelinian dichotomy: Going, going… but still not gone. The British Journal of Psychiatry, 196(2), 92–95. 10.1192/bjp.bp.109.073429 20118450 PMC2815936

[R7] Cross-Disorder Group of the Psychiatric Genomics Consortium. (2013). Identification of risk loci with shared effects on five major psychiatric disorders: A genome-wide analysis. Lancet (London, England), 381(9875), 1371–1379. 10.1016/S0140-6736(12)62129-1 23453885 PMC3714010

[R8] Cuthbert BN, & Insel TR (2013). Toward the future of psychiatric diagnosis: The seven pillars of RDoC. BMC Medicine, 11(1), 126. 10.1186/1741-7015-11-126 23672542 PMC3653747

[R9] Das S, Forer L, Schönherr S, Sidore C, Locke AE, Kwong A, Vrieze SI, Chew EY, Levy S, McGue M, Schlessinger D, Stambolian D, Loh P-R, Iacono WG, Swaroop A, Scott LJ, Cucca F, Kronenberg F, Boehnke M, … Fuchsberger C (2016). Next-generation genotype imputation service and methods. Nature Genetics, 48(10), 1284–1287. 10.1038/ng.3656 27571263 PMC5157836

[R10] de Jong K, Nugter MA, Polak MG, Wagenborg JEA, Spinhoven P, & Heiser WJ (2007). The Outcome Questionnaire (OQ-45) in a Dutch population: A cross-cultural validation. Clinical Psychology & Psychotherapy, 14(4), 288–301. 10.1002/cpp.529

[R11] de la Fuente J, Davies G, Grotzinger AD, Tucker-Drob EM, & Deary IJ (2021). A general dimension of genetic sharing across diverse cognitive traits inferred from molecular data. Nature human. Behaviour, 5(1), 49–58. 10.1038/s41562-020-00936-2 PMC9346507 32895543

[R12] Demontis D, Walters GB, Athanasiadis G, Walters R, Therrien K, Nielsen TT, Farajzadeh L, Voloudakis G, Bendl J, Zeng B, Zhang W, Grove J, Als TD, Duan J, Satterstrom FK, Bybjerg-Grauholm J, Bækved-Hansen M, Gudmundsson OO, Magnusson SH, … Børglum AD (2023). Genome-wide analyses of ADHD identify 27 risk loci, refine the genetic architecture and implicate several cognitive domains. Nature Genetics, 55(2), 198–208. 10.1038/s41588-022-01285-8 36702997 PMC10914347

[R13] Dudbridge F (2013). Power and predictive accuracy of polygenic risk scores. PLoS Genetics, 9(3), e1003348. 10.1371/journal.pgen.1003348 23555274 PMC3605113

[R14] Eijndhoven P. v., Collard R, Vrijsen J, Geurts DEM, Vasquez AA, Schellekens A, Munckhof E. v. d., Brolsma S, Duyser F, Bergman A, Oort J. v., Tendolkar I, & Schene A (2022). Measuring integrated novel dimensions in neurodevelopmental and stress-related mental disorders (MIND-SET): Protocol for a cross-sectional comorbidity study from a research domain criteria perspective. JMIRx Medicine, 3(1), e31269. 10.2196/31269 PMC10414459 37725542

[R15] Fairley S, Lowy-Gallego E, Perry E, & Flicek P (2020). The International Genome Sample Resource (IGSR) collection of open human genomic variation resources. Nucleic Acids Research, 48(D1), D941–D947. 10.1093/nar/gkz836 31584097 PMC6943028

[R16] First M, Gibbon M, Spitzer R, Williams J, & Benjamin L (1997). Structured clinical interview for DSM-IV-TR axis I disorders, research version, patient edition. (SCID-I/P). Biometrics Research, New York State Psychiatric Institute.

[R17] GBD 2016 DALYs and HALE Collaborators. (2017). Global, regional, and national disability-adjusted life-years (DALYs) for 333 diseases and injuries and healthy life expectancy (HALE) for 195 countries and territories, 1990–2016: A systematic analysis for the global burden of disease study 2016. Lancet (London, England), 390(10100), 1260–1344. 10.1016/S0140-6736(17)32130-X 28919118 PMC5605707

[R18] Ge T, Chen C-Y, Ni Y, Feng Y-CA, & Smoller JW (2019). Polygenic prediction via Bayesian regression and continuous shrinkage priors. Nature Communications, 10(1), 1776. 10.1038/s41467-019-09718-5 PMC6467998 30992449

[R19] Genetics of Personality Consortium. (2015). Meta-analysis of genome-wide association studies for neuroticism, and the polygenic association with major depressive disorder. JAMA Psychiatry, 72(7), 642–650. 10.1001/jamapsychiatry.2015.0554 25993607 PMC4667957

[R20] Grotzinger AD, Mallard TT, Akingbuwa WA, Ip HF, Adams MJ, Lewis CM, McIntosh AM, Grove J, Dalsgaard S, Lesch K-P, Strom N, Meier SM, Mattheisen M, Børglum AD, Mors O, Breen G, Lee PH, Kendler KS, Smoller JW, … Nivard MG (2022). Genetic architecture of 11 major psychiatric disorders at biobehavioral, functional genomic and molecular genetic levels of analysis. Nature Genetics, 54(5), 548–559. 10.1038/s41588-022-01057-4 35513722 PMC9117465

[R21] Grove J, Ripke S, Als TD, Mattheisen M, Walters RK, Won H, Pallesen J, Agerbo E, Andreassen OA, Anney R, Awashti S, Belliveau R, Bettella F, Buxbaum JD, Bybjerg-Grauholm J, Bækvad-Hansen M, Cerrato F, Chambert K, Christensen JH, … Børglum AD (2019). Identification of common genetic risk variants for autism spectrum disorder. Nature Genetics, 51(3), 431–444. 10.1038/s41588-019-0344-8 30804558 PMC6454898

[R22] Horn JL (1965). A rationale and test for the number of factors in factor analysis. Psychometrika, 30(2), 179–185. 10.1007/BF02289447 14306381

[R23] Howard DM, Adams MJ, Clarke T-K, Hafferty JD, Gibson J, Shirali M, Coleman JRI, Hagenaars SP, Ward J, Wigmore EM, Alloza C, Shen X, Barbu MC, Xu EY, Whalley HC, Marioni RE, Porteous DJ, Davies G, Deary IJ, … McIntosh AM (2019). Genome-wide meta-analysis of depression identifies 102 independent variants and highlights the importance of the prefrontal brain regions. Nature Neuroscience, 22(3), 343–352. 10.1038/s41593-018-0326-7 30718901 PMC6522363

[R24] Howard DM, Adams MJ, Shirali M, Clarke T-K, Marioni RE, Davies G, Coleman JRI, Alloza C, Shen X, Barbu MC, Wigmore EM, Gibson J, 23andMe Research Team, Hagenaars SP, Lewis CM, Ward J, Smith DJ, Sullivan PF, Haley CS, … McIntosh AM (2018). Genome-wide association study of depression phenotypes in UK biobank identifies variants in excitatory synaptic pathways. Nature Communications, 9(1), 1470. 10.1038/s41467-018-03819-3 PMC6117285 30166530

[R25] Kalia SS, Adelman K, Bale SJ, Chung WK, Eng C, Evans JP, Herman GE, Hufnagel SB, Klein TE, Korf BR, McKelvey KD, Ormond KE, Richards CS, Vlangos CN, Watson M, Martin CL, & Miller DT (2017). Recommendations for reporting of secondary findings in clinical exome and genome sequencing, 2016 update (ACMG SF v2. 0): A policy statement of the American College of Medical Genetics and Genomics. Genetics in Medicine, 19(2), 249–255. 27854360 10.1038/gim.2016.190

[R26] Kember RL, Merikangas AK, Verma SS, Verma A, Judy R, Regeneron Genetics Center, Damrauer SM, Ritchie MD, Rader DJ, & Bucan M (2021). Polygenic risk of psychiatric disorders exhibits cross-trait associations in electronic health record data from European ancestry individuals. Biological Psychiatry, 89(3), 236–245. 10.1016/j.biopsych.2020.06.026 32919613 PMC7770066

[R27] Khera AV, Chaffin M, Aragam KG, Haas ME, Roselli C, Choi SH, Natarajan P, Lander ES, Lubitz SA, Ellinor PT, & Kathiresan S (2018). Genome-wide polygenic scores for common diseases identify individuals with risk equivalent to monogenic mutations. Nature Genetics, 50(9), Article 9–Article 1224. 10.1038/s41588-018-0183-z PMC6128408 30104762

[R28] Klein Hofmeijer-Sevink M, Batelaan NM, van Megen HJGM, Penninx BW, Cath DC, van den Hout MA, & van Balkom AJLM (2012). Clinical relevance of comorbidity in anxiety disorders: A report from The Netherlands study of depression and anxiety (NESDA). Journal of Affective Disorders, 137(1), 106–112. 10.1016/j.jad.2011.12.008 22240085

[R29] Kooij JJS, & Francken MH (2010). Diagnostic interview for ADHD in adults (DIVA). DIVA Foundation.

[R30] Kotov R, Krueger RF, Watson D, Achenbach TM, Althoff RR, Bagby RM, Brown TA, Carpenter WT, Caspi A, Clark LA, Eaton NR, Forbes MK, Forbush KT, Goldberg D, Hasin D, Hyman SE, Ivanova MY, Lynam DR, Markon K, … Zimmerman M (2017). The hierarchical taxonomy of psychopathology (HiTOP): A dimensional alternative to traditional nosologies. Journal of Abnormal Psychology, 126(4), 454–477. 10.1037/abn0000258 28333488

[R31] Kozak MJ, & Cuthbert BN (2016). The NIMH research domain criteria initiative: Background, issues, and pragmatics. Psychophysiology, 53(3), 286–297. 10.1111/psyp.12518 26877115

[R32] Kretzschmar W, Mahajan A, Sharp K, McCarthy M, & Haplotype Reference Consortium. (2016). A reference panel of 64,976 haplotypes for genotype imputation. Nature Genetics, 48. 10.1038/ng.3643 PMC5388176 27548312

[R33] Lam M, Awasthi S, Watson HJ, Goldstein J, Panagiotaropoulou G, Trubetskoy V, Karlsson R, Frei O, Fan C-C, De Witte W, Mota NR, Mullins N, Brügger K, Lee SH, Wray NR, Skarabis N, Huang H, Neale B, Daly MJ, … Ripke S (2020). RICOPILI: Rapid imputation for COnsortias PIpeLIne. Bioinformatics, 36(3), 930–933. 10.1093/bioinformatics/btz633 31393554 PMC7868045

[R34] Lee PH, Anttila V, Won H, Feng Y-CA, Rosenthal J, Zhu Z, Tucker-Drob EM, Nivard MG, Grotzinger AD, Posthuma D, Wang MM-J, Yu D, Stahl EA, Walters RK, Anney RJL, Duncan LE, Ge T, Adolfsson R, Banaschewski T, … Smoller JW (2019). Genomic relationships, novel loci, and pleiotropic mechanisms across eight psychiatric disorders. Cell, 179(7), 1469–1482.e11. 10.1016/j.cell.2019.11.020 31835028 PMC7077032

[R35] Luykx JJ, Loef D, Lin B, van Diermen L, Nuninga JO, van Exel E, Oudega ML, Rhebergen D, Schouws SNTM, van Eijndhoven P, Verwijk E, Schrijvers D, Birkenhager TK, Ryan KM, Arts B, van Bronswijk SC, Kenis G, Schurgers G, Baune BT, … Rutten BPF (2022). Interrogating associations between polygenic liabilities and electroconvulsive therapy effectiveness. Biological Psychiatry, 91(6), 531–539. 10.1016/j.biopsych.2021.10.013 34955169

[R36] McCoy TH, Yu S, Hart KL, Castro VM, Brown HE, Rosenquist JN, Doyle AE, Vuijk PJ, Cai T, & Perlis RH (2018). High throughput phenotyping for dimensional psychopathology in electronic health records. Biological Psychiatry, 83(12), 997–1004. 10.1016/j.biopsych.2018.01.011 29496195 PMC5972065

[R37] Mitchell BL, Thorp JG, Wu Y, Campos AI, Nyholt DR, Gordon SD, Whiteman DC, Olsen CM, Hickie IB, Martin NG, Medland SE, Wray NR, & Byrne EM (2021). Polygenic risk scores derived from varying definitions of depression and risk of depression. JAMA Psychiatry, 78(10), 1152–1160. 10.1001/jamapsychiatry.2021.1988 34379077 PMC8358814

[R38] Mulders PCR, van Eijndhoven PFP, van Oort J, Oldehinkel M, Duyser FA, Kist JD, Collard RM, Vrijsen JN, Haak KV, Beckmann CF, Tendolkar I, & Marquand AF (2022). Striatal connectopic maps link to functional domains across psychiatric disorders. Translational Psychiatry, 12(1), 513. 10.1038/s41398-022-02273-6 36513630 PMC9747785

[R39] Mullins N, Forstner AJ, O’Connell KS, Coombes B, Coleman JRI, Qiao Z, Als TD, Bigdeli TB, Børte S, Bryois J, Charney AW, Drange OK, Gandal MJ, Hagenaars SP, Ikeda M, Kamitaki N, Kim M, Krebs K, Panagiotaropoulou G, … Andreassen OA (2021). Genome-wide association study of over 40,000 bipolar disorder cases provides new insights into the underlying biology. Nature Genetics, 53(6), 817–829. 10.1038/s41588-021-00857-4 34002096 PMC8192451

[R40] Overbeek T, Schruers K, & Griez E (2002). Comorbidity of obsessive-compulsive disorder and depression: Prevalence, symptom severity, and treatment effect. The Journal of Clinical Psychiatry, 63(12), 3395–1112. 10.4088/jcp.v63n1204 12523869

[R41] Plana-Ripoll O, Pedersen CB, Holtz Y, Benros ME, Dalsgaard S, de Jonge P, Fan CC, Degenhardt L, Ganna A, Greve AN, Gunn J, Iburg KM, Kessing LV, Lee BK, Lim CCW, Mors O, Nordentoft M, Prior A, Roest AM, … McGrath JJ (2019). Exploring comorbidity within mental disorders among a Danish National Population. JAMA Psychiatry, 76(3), 259–270. 10.1001/jamapsychiatry.2018.3658 30649197 PMC6439836

[R42] Purves KL, Coleman JRI, Meier SM, Rayner C, Davis KAS, Cheesman R, Bækvad-Hansen M, Børglum AD, Wan Cho S, Jürgen Deckert J, Gaspar HA, Bybjerg-Grauholm J, Hettema JM, Hotopf M, Hougaard D, Hübel C, Kan C, McIntosh AM, Mors O, … Eley TC (2020). A major role for common genetic variation in anxiety disorders. Molecular Psychiatry, 25(12), 3292–3303. 10.1038/s41380-019-0559-1 31748690 PMC7237282

[R43] Schippers GM, Broekman TG, Buchholz A, Koeter MWJ, & van den Brink W (2010). Measurements in the addictions for triage and evaluation (MATE): An instrument based on the World Health Organization family of international classifications. Addiction (Abingdon, England), 105(5), 862–871. 10.1111/j.1360-0443.2009.02889.x 20331557

[R44] The Brainstorm Consortium, Anttila V, Bulik-Sullivan B, Finucane HK, Walters RK, Bras J, Duncan L, Escott-Price V, Falcone GJ, Gormley P, Malik R, Patsopoulos NA, Ripke S, Wei Z, Yu D, Lee PH, Turley P, Grenier-Boley B, Chouraki V, … Neale BM (2018). Analysis of shared heritability in common disorders of the brain. Science, 360(6395), eaap8757. 10.1126/science.aap8757 29930110 PMC6097237

[R45] Trubetskoy V, Pardiñas AF, Qi T, Panagiotaropoulou G, Awasthi S, Bigdeli TB, Bryois J, Chen C-Y, Dennison CA, Hall LS, Lam M, Watanabe K, Frei O, Ge T, Harwood JC, Koopmans F, Magnusson S, Richards AL, Sidorenko J, … O’Donovan MC (2022). Mapping genomic loci implicates genes and synaptic biology in schizophrenia. Nature, 604(7906), 502–508. 10.1038/s41586-022-04434-5 35396580 PMC9392466

[R46] Vuijk R (2016). Nederlands interview ten behoeve van diagnostiek autism-spectrumstoornis bij volwassenen (NIDA). Sarr Expertisecentrum Autisme/Dare to Design.

[R47] Wenzel A (2017). World Health Organization Disability Assessment Schedule 2.0. In Wenzel A (Ed.), The SAGE encyclopedia of abnormal and clinical psychology. SAGE Publications, Inc.. 10.4135/9781483365817.n1493

[R48] Wolfers T, Doan NT, Kaufmann T, Alnæs D, Moberget T, Agartz I, Buitelaar JK, Ueland T, Melle I, Franke B, Andreassen OA, Beckmann CF, Westlye LT, & Marquand AF (2018). Mapping the heterogeneous phenotype of schizophrenia and bipolar disorder using normative models. JAMA Psychiatry, 75(11), 1146–1155. 10.1001/jamapsychiatry.2018.2467 30304337 PMC6248110

[R49] World Health Organization. (1993). ICD-10: The ICD-10 Classification of Mental and Behavioural Disorders: Diagnostic criteria for research (p. xiii–248). World Health Organization.

[R50] Wray NR, Ripke S, Mattheisen M, Trzaskowski M, Byrne EM, Abdellaoui A, Adams MJ, Agerbo E, Air TM, Andlauer TMF, Bacanu S-A, Bækvad-Hansen M, Beekman AFT, Bigdeli TB, Binder EB, Blackwood DRH, Bryois J, Buttenschøn HN, Bybjerg-Grauholm J, … Sullivan PF (2018). Genome-wide association analyses identify 44 risk variants and refine the genetic architecture of major depression. Nature Genetics, 50(5), 668–681. 10.1038/s41588-018-0090-3 29700475 PMC5934326

[R51] Zimmerman M, Ellison W, Young D, Chelminski I, & Dalrymple K (2015). How many different ways do patients meet the diagnostic criteria for major depressive disorder? xComprehensive Psychiatry, 56, 29–34. 10.1016/j.comppsych.2014.09.007 25266848

