# Supplementary material for: Multi-polygenic scores in psychiatry: From disorder specific to transdiagnostic perspectives
Source: Am J Med Genet B Neuropsychiatr Genet. Author manuscript; Available in PMC 2025 Jan 1. (PMC10803201; doi:10.1002/ajmg.b.32951)
Supplement: Supplementary materials [file NIHMS1952897-supplement-Supplementary_materials.docx]

Supplementary materials for

**Multi-polygenic scores in psychiatry: from disorder-specific to transdiagnostic perspectives**

Yingjie Shi^1,2^, Emma Sprooten^1,2,3^, Peter Mulders^2,4^, Janna Vrijsen^2,4,5^, Janita Bralten^1,2^, Ditte Demontis^6,7,8^, Anders D. Børglum^6,7,8^, G. Bragi Walters^9,10^, Kari Stefansson^9,10^, Philip van Eijndhoven^2,4^, Indira Tendolkar^2,4^, Barbara Franke^1,2,4^, Nina Roth Mota^1,2^

1 Department of Human Genetics, Radboud University Medical Center, Nijmegen, The Netherlands

2 Donders Institute for Brain, Cognition and Behaviour, Radboud University, Nijmegen, The Netherlands

3 Department of Cognitive Neuroscience, Radboud University Medical Center, Nijmegen, The Netherlands

4 Department of Psychiatry, Radboud University Medical Center, Nijmegen, The Netherlands

5 Pro Persona Mental Health Care, Depression Expertise Centre, Nijmegen, The Netherlands

6 Department of Biomedicine/Human Genetics, Aarhus University, Aarhus, Denmark

7 The Lundbeck Foundation Initiative for Integrative Psychiatric Research, iPSYCH, Denmark

8 Center for Genomics and Personalized Medicine, Aarhus, Denmark

9 deCODE Genetics, Reykjavík, Iceland

10 Faculty of Medicine, University of Iceland, Reykjavík, Iceland

**Table S1. Overview of diagnosis and grouping in the MIND-SET cohort.**

| **Disorder group** | **Diagnosis** | **Sample size** | |
| --- | --- | --- | --- |
| **Mood Disorders** | Past unipolar depression | 360 | 276 |
|  | Current dysthymia |  | 54 |
|  | Current depressive episode |  | 195 |
|  | Current (hypo)manic episode |  | 4 |
|  | Bipolar disorder |  | 21 |
| **Anxiety Disorders** | Panic disorder | 147 | 33 |
|  | Agoraphobia |  | 4 |
|  | Social phobia |  | 44 |
|  | Specific phobia |  | 11 |
|  | Obsessive-compulsive disorder |  | 16 |
|  | Post-traumatic stress disorder |  | 35 |
|  | Generalized anxiety disorder |  | 28 |
|  | Anxiety not otherwise specified |  | 18 |
| **ADHD** | Attention-deficit/hyperactivity disorder | 171 | |
| **ASD** | Autism spectrum disorder | 121 | |
| **Substance Use Disorders** | Alcohol | 121 | 35 |
|  | Nicotine |  | 76 |
|  | Cannabis |  | 23 |
|  | Opioids |  | 4 |
|  | Cocaine |  | 2 |
|  | Stimulants |  | 6 |
|  | Sedatives |  | 10 |
|  | Other drugs |  | 2 |
|  | Gambling |  | 1 |

**Table S2. Demographic information for unaffected and affected groups in the MIND-SET cohort.**

|  |  | Unaffected | Affected | | | | | |
| --- | --- | --- | --- | --- | --- | --- | --- | --- |
|  |  |  | Total | Mood disorders | Anxiety disorders | ADHD | ASD | Substance use disorders |
| Gender | Female | 32 | 205 | 169 | 72 | 72 | 41 | 41 |
|  | Male | 29 | 247 | 191 | 75 | 99 | 80 | 80 |
| Mean age (SD) |  | 39.36 (16.28) | 40.13 (14.26) | 40.63 (14.51) | 39.61 (13.77) | 36.27 (11.71) | 36.27 (12.87) | 41.31 (14.33) |
| Level of education  (%) | Higher education | 42  (69%) | 182  (40%) | 145  (40%) | 60  (41%) | 57  (33%) | 57  (47%) | 37  (31%) |
|  | Secondary education | 14  (23%) | 168  (37%) | 136  (38%) | 46  (31%) | 74  (43%) | 46  (38%) | 51  (42%) |
|  | Primary education | 5  (8%) | 75  (17%) | 58  (16%) | 28  (19%) | 26  (15%) | 15  (12%) | 21  (17%) |
|  | No education | 0  (0%) | 4  (1%) | 3  (1%) | 4  (3%) | 1  (1%) | 1  (1%) | 1  (1%) |
|  | Missing value | 0  (0%) | 23  (5%) | 18  (5%) | 9  (6%) | 13  (8%) | 2  (2%) | 11  (9%) |

***Note*.** There were no significant gender or age differences (Chi-squared = 1.092, *p* = .296; t = -0.391, *p* = .696) between the affected and unaffected groups, or between any disorder categories and the unaffected control group. However, the affected group in general had lower levels of education than the unaffected comparison group (Chi-squared = 15.327, *p* = .002), and significant differences in education levels were also present in the individual groups with mood disorders (Chi-squared = 14.818 , *p* = .002), ADHD (Chi-squared = 19.213, *p* = 2e-4), and SUD (Chi-squared = 19.810, *p* = 2e-4) compared to the unaffected control group. Therefore, we provided additional test statistics after removing the effect of levels of education (**Table S9**).

**Table S3. Questionnaire subscales included in factor analysis.**

| Symptoms/ Traits | Questionnaires | Subscales | |
| --- | --- | --- | --- |
| Depressive symptoms | Inventory of Depressive Symptomatology -Self Rating (IDS-SR) [1] | No subscales | |
| Anxiety sensitivity | Anxiety Sensitivity Index (ASI) [2] | No subscales | |
| ADHD symptom severity | Conners’ Adult ADHD Rating Scale (CAARS) [3] | Inattention/memory problems  Hyperactivity/restlessness  Impulsivity/emotional liability  Problems with self-concept | |
| Autistic traits | Autism Spectrum Quotient-50 (AQ-50) [4] | Social insight and behavior  Switching attention/difficulty with changes  Communication  Fantasy/Imagination  Detail-oriented | |
| Personality traits | Personality Inventory for DSM-5-Short Form (PID-5-SF) [5] | Negative affect  Detachment  Antagonism  Disinhibition  Psychoticism | |
| Psychological constructs:   - Alexithymia - Behavioral regulation - Repetitive thoughts | Toronto Alexithymia Scale-20 (TAS-20) [6] | Difficulty describing feelings  Difficulty identifying feelings  Externally-oriented thinking | |
|  | Behavior Rating Inventory Executive Function – Adult (BRIEF-A) [7] | Inhibit  Shift  Emotional Control  Self-Monitor  Initiate | Working Memory  Plan/Organise  Organisation of Materials  Task Monitor |
|  | Perseverative Thinking Questionnaire (PTQ) [8] | Repetitiveness  Intrusiveness  difficulties to disengage | |

**Table S4. Overview of the sources of PRS base GWASs.**

|  | **Phenotype** | **Abbreviation** | **Cohort** | **#Cases** | **#Controls** | **#Total** | **h^2^_SNP_ (SE)** |
| --- | --- | --- | --- | --- | --- | --- | --- |
| 1 | Anxiety disorders [9] | **ANX** | UKBB, iPSYCH, ANGST | 25,453 | 58,113 | 83,566 | 0.260 (0.011) |
| 2 | Attention-deficit/hyperactivity disorder [10] | ADHD | iPSYCH + 10 PGC cohorts + deCODE | 38,691 | 186,843 | 225,534 | 0.140 (0.01) |
| 3 | Autism spectrum disorder [11] | ASD | 5 PGC trio samples + iPSYCH | 18,382 | 27,969 | 46,351 | 0.118 (0.010) |
| 4 | Bipolar disorder [12] | BP | PGC 57 cohorts | 41,917 | 371,549 | 413,466 | 0.186 (0.008) |
| 5 | Schizophrenia [13] | SCZ | PGC3 | 53,386 | 77,258 | 130,644 | 0.240 (0.007) |
| 6 | Major depressive disorder [14] | MDD | 29 PGC MDD2 cohorts, deCODE, Generation Scotland, GERA, and iPSYCH cohorts | 45,396 | 97,250 | 142,646 | 0.087 (0.004) |
| 7 | Broadly defined depression [15] | DEP | 33 PGC cohorts + broad depression phenotype in UKB | 170,756 | 329,443 | 500,199 | 0.089 (0.003) |
| 8 | Subset broadly defined depression from UKB [16] | subset-DEP | Broad depression phenotype in UKB | 113,769 | 208,811 | 322,580 | 0.102 (0.004) |
| 9 | Eight psychiatric disorders: MDD, ADHD, ASD, BP, SCZ, anorexia nervosa, obsessive-compulsive disorder, Tourette syndrome [17] | Cross-disorder | Aggregated from eight GWASs | 162,151 | 276,846 | 438,997 | - |

h^2^_SNP_ – SNP heritability; SE – standard error.

**Table S5. Power estimates of PRSs with their corresponding phenotype in the target cohort.**

|  | **ANX-PRS** | **ADHD-PRS** | **ASD-PRS** | **MDD-PRS** | **DEP-PRS** |
| --- | --- | --- | --- | --- | --- |
| **target phenotype** | **Anxiety disorders** | **ADHD** | **ASD** | **Mood disorders** | **Mood disorders** |
| **nsnp** | 116587 | 176994 | 131901 | 133351 | 221396 |
| **n_discovery** | 83566 | 225534 | 46351 | 142646 | 500199 |
| **n_target** | 208 | 232 | 182 | 421 | 421 |
| **vg1** | 0.260 | 0.140 | 0.120 | 0.100 | 0.100 |
| **cov12** | 0.260 | 0.140 | 0.120 | 0.100 | 0.087 |
| **pi0** | 0.950 | 0.950 | 0.950 | 0.950 | 0.950 |
| **prevalence_discovery** | 0.200 | 0.050 | 0.012 | 0.150 | 0.350 |
| **prevalence_target** | 0.200 | 0.050 | 0.012 | 0.150 | 0.080 |
| **sampling_discovery** | 0.160 | 0.170 | 0.400 | 0.280 | 0.350 |
| **sampling_target** | 0.710 | 0.737 | 0.660 | 0.850 | 0.860 |
| **power_bestPt** | 0.515 | 0.741 | 0.455 | 0.276 | 0.538 |

***Note.*** nsnp - number of independent markers (after LD clumping) in the polygenic score; vg1 – SNP heritability of trait in base GWAS; cov12 - covariance between genetic effect sizes in the two samples; pi0 - proportion of markers with no effect on the training trait; power_bestPt – power estimates of the best fitting p threshold. The estimates of SNP heritability, genetic correlations, and population prevalence from the original GWAS publication. For ANX, ADHD, ASD, and MDD, we assumed the same phenotypes in base and target cohorts (i.e., the same SNP heritability, genetic correlation equals 1). For the association between DEP-PRS and mood disorders, the genetic correlation was obtained from [15].

**Table S6. Full test statistics of associations between PRSs and disorder diagnoses.**

| Base disorder | Phenotype | Threshold | PRS.R^2^ | Full.R^2^ | Null.R^2^ | Adj.R^2^ | Coefficient | StandardError | *p* | Num.SNP | Empirical-P |
| --- | --- | --- | --- | --- | --- | --- | --- | --- | --- | --- | --- |
| ANX-PRS | MoodDis | 0.0001 | 0.035 | 0.054 | 0.02 | 0.045 | 0.403 | 0.141 | .004 | 198 | .027 |
|  | AnxDis | 0.0001 | 0.039 | 0.073 | 0.034 | 0.04 | 0.4 | 0.168 | .017 | 198 | .104 |
|  | ADHD | 0.0001 | 0.048 | 0.098 | 0.05 | 0.037 | 0.455 | 0.165 | .006 | 198 | .042 |
|  | ASD | 0.0001 | 0.039 | 0.141 | 0.101 | 0.02 | 0.375 | 0.162 | .020 | 198 | .115 |
|  | SUD | 0.0001 | 0.06 | 0.14 | 0.08 | 0.064 | 0.482 | 0.17 | .005 | 198 | .030 |
| ADHD-PRS | MoodDis | 0.01 | 0.013 | 0.033 | 0.02 | 0.018 | 0.257 | 0.144 | .075 | 10471 | .285 |
|  | AnxDis | 0.01 | 0.016 | 0.051 | 0.034 | 0.017 | 0.267 | 0.173 | .122 | 10471 | .438 |
|  | ADHD | 0.01 | 0.047 | 0.097 | 0.05 | 0.036 | 0.453 | 0.166 | .006 | 10471 | .034 |
|  | ASD | 0.01 | 0.033 | 0.134 | 0.101 | 0.016 | 0.367 | 0.174 | .035 | 10471 | .157 |
|  | SUD | 0.01 | 0.03 | 0.11 | 0.08 | 0.032 | 0.341 | 0.17 | .044 | 10471 | .185 |
| ASD-PRS | MoodDis | 0.0001 | 0.01 | 0.03 | 0.02 | 0.013 | -0.217 | 0.14 | .120 | 198 | .525 |
|  | AnxDis | 1 | 0.003 | 0.038 | 0.034 | 0.003 | 0.107 | 0.155 | .489 | 131901 | .981 |
|  | ADHD | 1 | 0.008 | 0.058 | 0.05 | 0.006 | 0.18 | 0.157 | .251 | 131901 | .804 |
|  | ASD | 0.001 | 0.006 | 0.107 | 0.101 | 0.003 | 0.15 | 0.164 | .358 | 1072 | .920 |
|  | SUD | 0.0001 | 0.022 | 0.102 | 0.08 | 0.023 | -0.264 | 0.152 | .083 | 198 | .412 |
| BP-PRS | MoodDis | 0.1 | 0.02 | 0.04 | 0.02 | 0.026 | 0.306 | 0.142 | .031 | 42102 | .117 |
|  | AnxDis | 0.2 | 0.041 | 0.075 | 0.034 | 0.042 | 0.383 | 0.156 | .014 | 64919 | .062 |
|  | ADHD | 0.05 | 0.025 | 0.075 | 0.05 | 0.019 | 0.316 | 0.159 | .046 | 27343 | .181 |
|  | ASD | 0.05 | 0.019 | 0.12 | 0.101 | 0.009 | 0.291 | 0.181 | .107 | 27343 | .363 |
|  | SUD | 0.01 | 0.033 | 0.113 | 0.08 | 0.035 | 0.354 | 0.168 | .034 | 10193 | .139 |
| SCZ-PRS | MoodDis | 0.01 | 0.026 | 0.046 | 0.02 | 0.035 | 0.348 | 0.139 | .012 | 13118 | .045 |
|  | AnxDis | 0.01 | 0.043 | 0.077 | 0.034 | 0.044 | 0.399 | 0.16 | .013 | 13118 | .043 |
|  | ADHD | 0.01 | 0.034 | 0.083 | 0.05 | 0.026 | 0.38 | 0.164 | .020 | 13118 | .070 |
|  | ASD | 0.0001 | 0.029 | 0.13 | 0.101 | 0.014 | 0.338 | 0.168 | .044 | 1900 | .122 |
|  | SUD | 0.01 | 0.024 | 0.105 | 0.08 | 0.026 | 0.303 | 0.166 | .068 | 13118 | .195 |
| MDD-PRS | MoodDis | 1 | 0.021 | 0.041 | 0.02 | 0.028 | 0.34 | 0.152 | .025 | 133351 | .121 |
|  | AnxDis | 0.0001 | 0.038 | 0.073 | 0.034 | 0.04 | 0.388 | 0.164 | .018 | 300 | .096 |
|  | ADHD | 0.0001 | 0.025 | 0.075 | 0.05 | 0.019 | 0.33 | 0.163 | .043 | 300 | .195 |
|  | ASD | 0.0001 | 0.023 | 0.124 | 0.101 | 0.011 | 0.311 | 0.174 | .073 | 300 | .305 |
|  | SUD | 1 | 0.025 | 0.105 | 0.08 | 0.026 | 0.328 | 0.178 | .066 | 133351 | .283 |
| DEP-PRS | MoodDis | 0.01 | 0.091 | 0.111 | 0.02 | 0.117 | 0.679 | 0.149 | 5e-6 | 12501 | 2e-4 |
|  | AnxDis | 0.01 | 0.133 | 0.168 | 0.034 | 0.138 | 0.732 | 0.172 | 2e-5 | 12501 | 5e-4 |
|  | ADHD | 0.01 | 0.13 | 0.179 | 0.05 | 0.101 | 0.749 | 0.169 | 9e-6 | 12501 | 1e-4 |
|  | ASD | 0.05 | 0.063 | 0.165 | 0.101 | 0.032 | 0.491 | 0.169 | .004 | 36093 | .021 |
|  | SUD | 0.01 | 0.132 | 0.212 | 0.08 | 0.141 | 0.763 | 0.187 | 4e-5 | 12501 | 2e-4 |
| Subset-DEP-PRS | MoodDis | 0.05 | 0.056 | 0.076 | 0.02 | 0.073 | 0.526 | 0.145 | 3e-4 | 34326 | .002 |
|  | AnxDis | 0.05 | 0.109 | 0.143 | 0.034 | 0.113 | 0.673 | 0.173 | 1e-4 | 34326 | .001 |
|  | ADHD | 0.05 | 0.077 | 0.127 | 0.05 | 0.059 | 0.579 | 0.167 | .001 | 34326 | .005 |
|  | ASD | 0.001 | 0.039 | 0.141 | 0.101 | 0.019 | 0.421 | 0.182 | .020 | 2272 | .100 |
|  | SUD | 0.05 | 0.057 | 0.138 | 0.08 | 0.061 | 0.502 | 0.181 | .006 | 34326 | .032 |
| Cross-PRS | MoodDis | 0.1 | 0.032 | 0.052 | 0.02 | 0.042 | 0.404 | 0.146 | .006 | 36990 | .032 |
|  | AnxDis | 0.1 | 0.052 | 0.086 | 0.034 | 0.053 | 0.441 | 0.162 | .006 | 36990 | .036 |
|  | ADHD | 0.01 | 0.034 | 0.084 | 0.05 | 0.026 | 0.365 | 0.157 | .020 | 10637 | .102 |
|  | ASD | 0.0001 | 0.03 | 0.132 | 0.101 | 0.015 | 0.336 | 0.163 | .039 | 1345 | .173 |
|  | SUD | 0.1 | 0.04 | 0.12 | 0.08 | 0.043 | 0.386 | 0.166 | .020 | 36990 | .103 |

***Note*.** The prevalence rates used to adjust the ascertainment bias for mood disorders, anxiety disorders, ADHD, ASD, and SUD were 0.15, 0.16, 0.05, 0.01, and 0.2, respectively. Adj.R^2^ – R^2^ on the liability scale adjusted for ascertainment bias.

**Table S7. Variance explained (pseudo-R^2^ [*p_emp_* values]) in diagnosis status by PRSs computed using PRS-CS-auto for different psychiatric disorders.** The proportion of variance explained by each PRS in each of the five psychiatric disorder diagnoses was estimated by Nagelkerke’s pseudo-R^2^, computed as the difference between the R^2^ of the single PRS model, containing one PRS and the covariates (i.e., age, sex, and four PCs), and the R^2^ of the null model, containing only the covariates.

| **Phenotype** | **ANX-PRS** | **ADHD-PRS** | **ASD-PRS** | **BP-PRS** | **SCZ-PRS** | **MDD-PRS** | **DEP-PRS** | **Cross-PRS** |
| --- | --- | --- | --- | --- | --- | --- | --- | --- |
| **MoodDis** | *0.017* | 0.012 | 2e-4 | 0.012 | 0.011 | *0.029* | **0.058** | 0.012 |
|  | *[.045]* | [.092] | [.830] | [.084] | [.106] | *[.009]* | **[2e-4]** | [.087] |
| **AnxDis** | *0.033* | 0.018 | 0.001 | 0.024 | *0.033* | *0.074* | **0.116** | *0.030* |
|  | *[.028]* | [.104] | [.718] | [.060] | *[.028]* | *[.001]* | **[7e-5]** | *0.037]* |
| **ADHD** | 0.020 | *0.029* | 0.002 | *0.029* | 0.016 | *0.028* | **0.072** | 0.011 |
|  | [.073] | *[.030]* | [.607] | *[.031]* | [.109] | *[.035]* | **[8e-4]** | [.184] |
| **ASD** | 0.027 | 0.007 | 0.001 | *0.020* | 0.025 | *0.027* | *0.033* | 0.012 |
|  | [.053] | [.315] | [.678] | *[.091]* | [.063] | *[.050]* | *[.033]* | [.192] |
| **SUD** | 0.026 | 0.028 | 0.000 | 0.017 | 0.008 | *0.048* | **0.094** | 0.011 |
|  | [.060] | [.053] | [.858] | [.129] | [.298] | *[.011]* | **[5e-4]** | [.214] |

***Note***. Associations that exceeded Bonferroni-corrected threshold of *p* = .001 were labeled in **bold**, and those exceeding the uncorrected threshold of *p* = .05 were labeled in *italic*. ANX - anxiety disorders; ADHD - attention-deficit/hyperactivity disorder; ASD - autism spectrum disorders; BP – bipolar disorder; SCZ – schizophrenia; MDD - major depressive disorder; DEP – depression; SUD – substance use disorders.

**Table S8. Full test statistics of associations between DEP-PRS and factor scores.**

| **Phenotype** | **Threshold** | **PRS.R^2^** | **Full.R^2^** | **Null.R^2^** | **Coefficient** | **Standard,Error** | ***p*** | **Num_SNP** | **Empirical-p** |
| --- | --- | --- | --- | --- | --- | --- | --- | --- | --- |
| F1 | 0.01 | 0.042 | 0.053 | 0.011 | 0.200 | 0.049 | 6e-5 | 12501 | 5e-4 |
| F2 | 0.2 | 0.029 | 0.085 | 0.056 | 0.169 | 0.049 | .001 | 91288 | .004 |
| F3 | 0.01 | 0.001 | 0.013 | 0.012 | 0.033 | 0.050 | .507 | 12501 | .960 |
| F4 | 0.001 | 0.018 | 0.023 | 0.005 | 0.134 | 0.050 | .008 | 3029 | .040 |

**Table S9. Sensitivity analysis of DEP-PRS including levels of education as a covariate.**

| Phenotype | Threshold | PRS.R^2^ | Full.R^2^ | Null.R^2^ | | Coefficient | Standard,Error | *p* | Num_SNP | Empirical-p |
| --- | --- | --- | --- | --- | --- | --- | --- | --- | --- | --- |
| MoodDis | 0.01 | 0.085 | 0.178 | 0.093 | 0.704 | | 0.164 | 2e-4 | 12501 | 1e-4 |
| AnxDis | 0.01 | 0.101 | 0.226 | 0.125 | 0.673 | | 0.183 | 2e-4 | 12501 | .001 |
| ADHD | 0.01 | 0.114 | 0.278 | 0.165 | 0.766 | | 0.186 | 4e-4 | 12501 | 5e-4 |
| ASD | 0.05 | 0.062 | 0.185 | 0.123 | 0.511 | | 0.182 | .005 | 36093 | .028 |
| SUD | 0.01 | 0.129 | 0.343 | 0.214 | 0.859 | | 0.215 | 7e-4 | 12501 | .001 |
|  |  |  |  |  |  | |  |  |  |  |
| F1 | 0.01 | 0.041 | 0.081 | 0.041 | 0.201 | | 0.051 | 9e-4 | 12501 | .001 |
| F2 | 0.2 | 0.030 | 0.114 | 0.083 | 0.174 | | 0.050 | .001 | 91288 | .003 |
| F3 | 0.01 | 0.003 | 0.026 | 0.023 | 0.052 | | 0.053 | .325 | 12501 | .806 |
| F4 | 0.001 | 0.022 | 0.077 | 0.055 | 0.148 | | 0.051 | .004 | 3029 | .020 |

**Table S10. Comparing generalized linear regression models with multi-PRSs and with single predictor of DEP-PRS.**

Model 1: Outcome ~ ANX.PRS + ADHD.PRS + ASD.PRS + BP.PRS + SCZ.PRS + MDD.PRS + DEP.PRS + Cross-disorder.PRS + PC1 + PC2 + PC3 + PC4 + Age + Sex

Model 2: Outcome ~ DEP.PRS + PC1 + PC2 + PC3 + PC4 + Age + Sex

| **Outcome** | **Df** | **Deviance** | ***p*** |
| --- | --- | --- | --- |
| **Mood disorders** | 7 | 9.297 | .232 |
| **Anxiety disorders** | 7 | 4.374 | .736 |
| **ADHD** | 7 | 8.434 | .296 |
| **ASD** | 7 | 7.633 | .366 |
| **SUD** | 7 | 11.820 | .107 |

*Note.* The variable inflation factors were below 4 for all PRS predictors and were below 2 for DEP-PRSs in all models, suggesting that the model estimates were unlikely to be biased by multicollinearity. PC1-PC4 – the first four (post-imputation) principal components derived from the best guess genotypes.

**Table S11. Multiple linear regression of individuals’ factor loadings on OQ-45 and WHODAS 2.0 scales.**

Linear hypotheses: OQ ~ F1 + F2 + F3 + F4

| **OQ** | **Estimate** | **SE** | **t value** | ***p*** |
| --- | --- | --- | --- | --- |
| **F1** | 0.762 | 0.027 | 28.396 | < 2e-16 |
| **F2** | 0.121 | 0.024 | 4.945 | 1e-6 |
| **F3** | 0.070 | 0.024 | 2.955 | .003 |
| **F4** | 0.123 | 0.026 | 4.686 | 4e-6 |

Linear hypotheses: WHODAS ~ F1 + F2 + F3 + F4

| **WHODAS** | **Estimate** | **SE** | **t value** | ***p*** |
| --- | --- | --- | --- | --- |
| **F1** | 0.643 | 0.036 | 18.037 | < 2e-16 |
| **F2** | 0.145 | 0.032 | 4.485 | 1e-5 |
| **F3** | 0.172 | 0.031 | 5.487 | 7e-8 |
| **F4** | 0.078 | 0.035 | 2.250 | .025 |

**Table S12. T-test statistics for factor loadings of patients versus controls.**

|  | **t value** | ***p*** | 95% CI |
| --- | --- | --- | --- |
| **F1** | -16.253 | <2e-16 | [-1.97, -1.55] |
| **F2** | -9.459 | <2e-16 | [-1.45, -0.95] |
| **F3** | -7.335 | 1e-12 | [-1.23, -0.71] |
| **F4** | -9.551 | <2-16 | [-1.46, -0.96] |


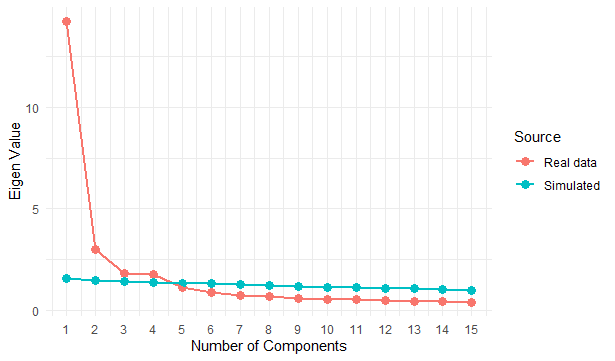


**Figure S1. Scree plot from parallel analysis.** Simulated eigenvalues were calculated from 500 randomly generated correlation matrices, which were then compared with eigenvalues extracted from the real data [18]. The 95th percentile of eigenvalues were used for this comparison, and the four factors larger than the simulated eigenvalues were retained. This analysis was performed using an R-based engine [19].

**
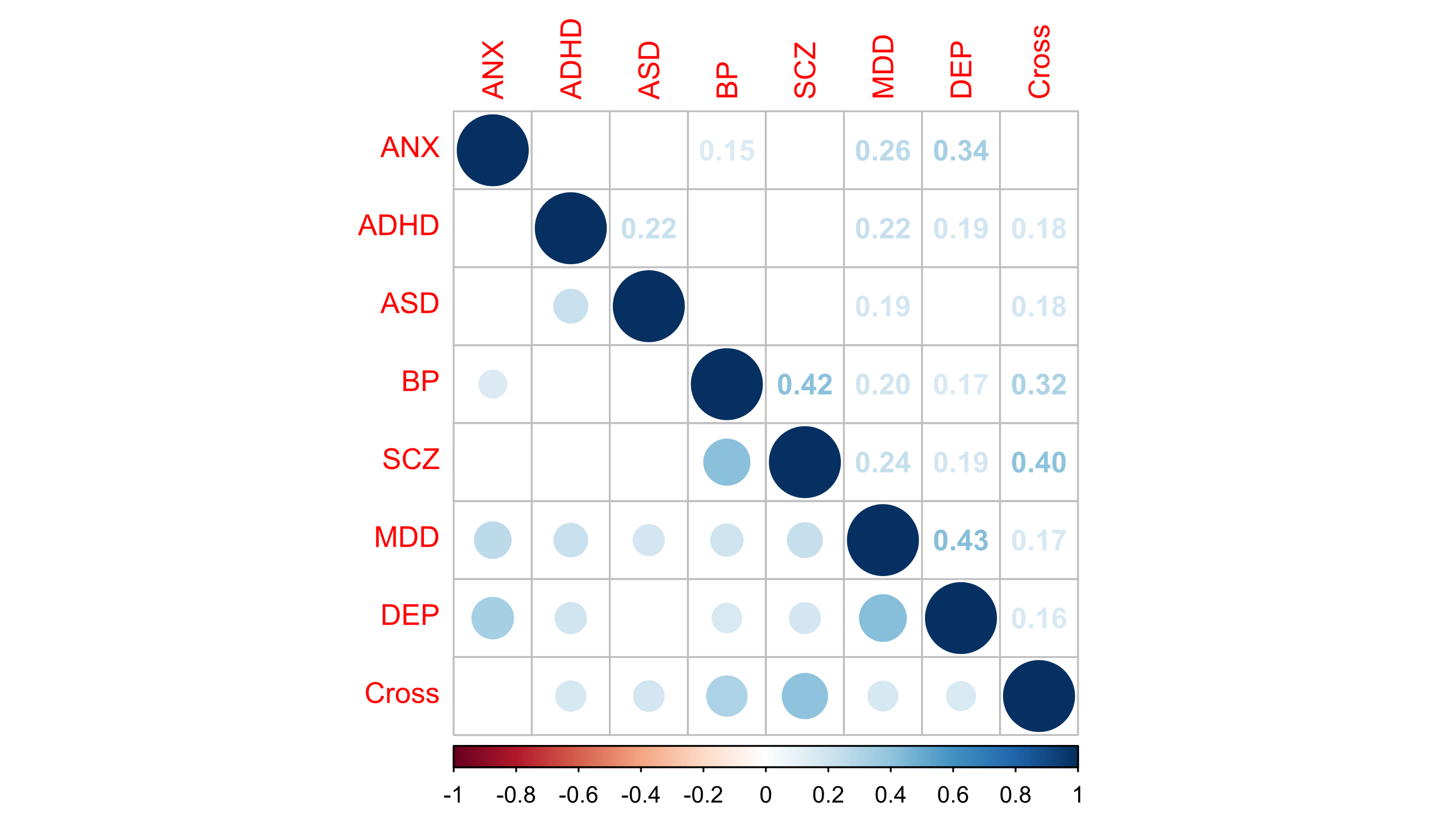
**

**Figure S2. Pairwise Pearson’s correlations among different disorder PRSs calculated at genome-wide level (P_T_ = 1).** Correlation coefficients are colored according to the value, as shown on the upper triangular. Insignificant correlations (p < .001) are left blank.

**
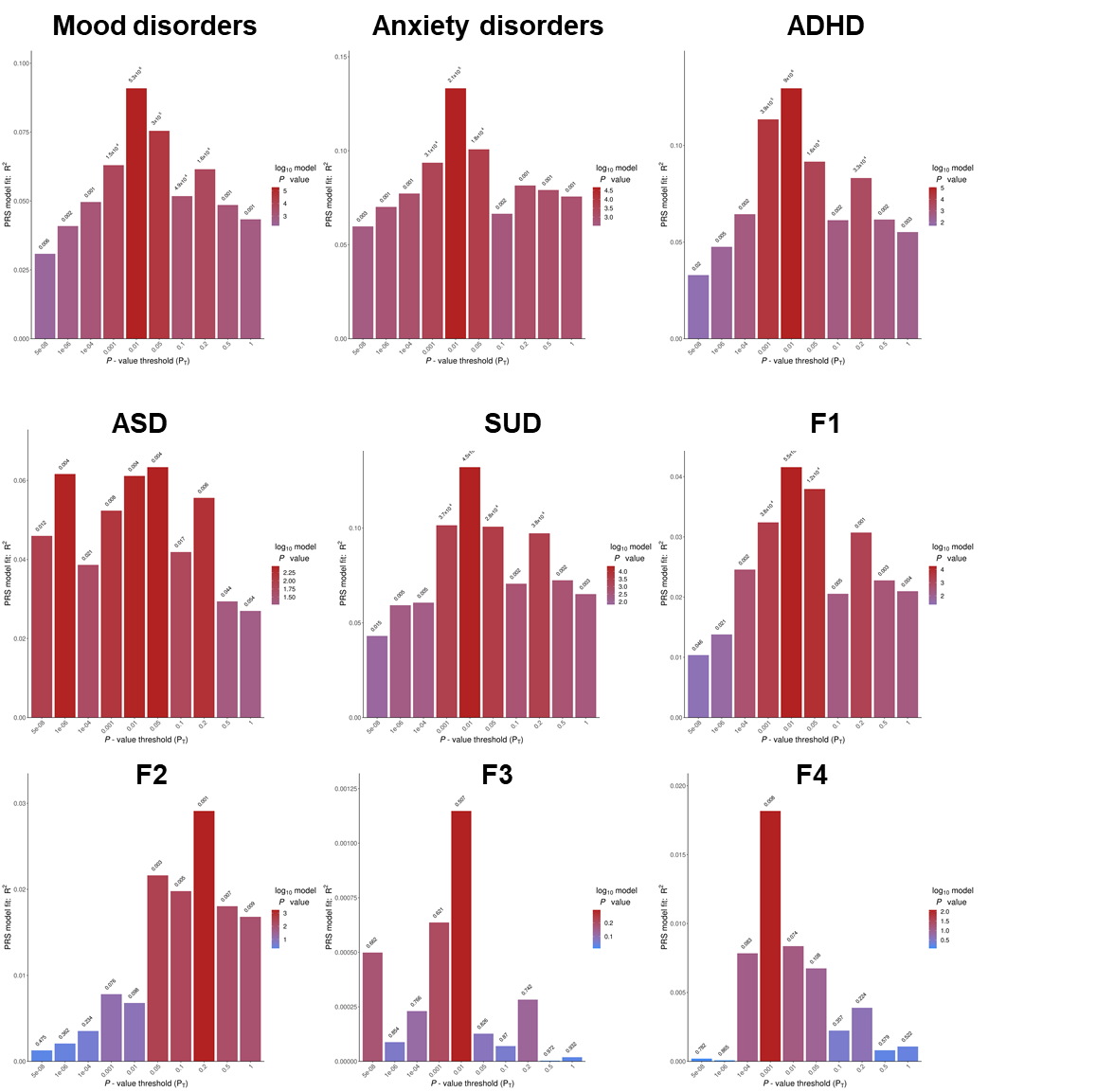
**

**Figure S3.** **Model fit of DEP-PRS at all *a priori* defined p-value thresholds.** In the association analyses for each diagnostic status or transdiagnostic factor, we chose the *p*-value threshold that yielded the highest R^2^. The chosen threshold is the same (Pt = 0.01) across all phenotypes that were significantly associated with DEP-PRS (Mood disorders, Anxiety disorders, ADHD, SUD, and F1). F1 - negative valence systems; F2 - social processes; F3 - cognitive systems; F4 - arousal/regulatory systems.

**
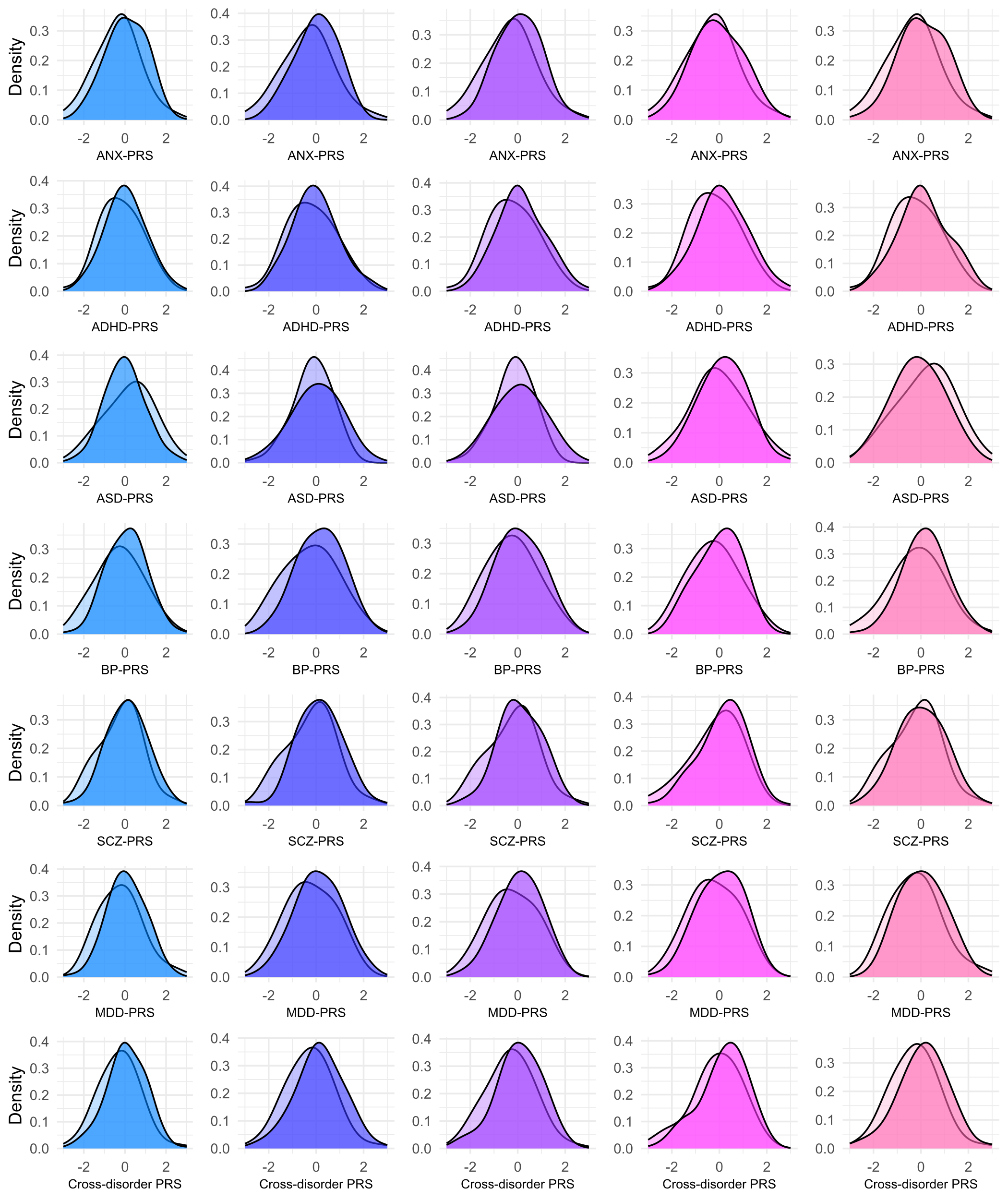
**

**Figure S4**. **PRS distributions for unaffected (light color) and affected individuals (dark color).** Each row shows the liability computed per base GWAS (denoted in the x-axis label). Each column shows the PRS distributions of individuals with or without the diagnosis per disorder (denoted as the column header).

**
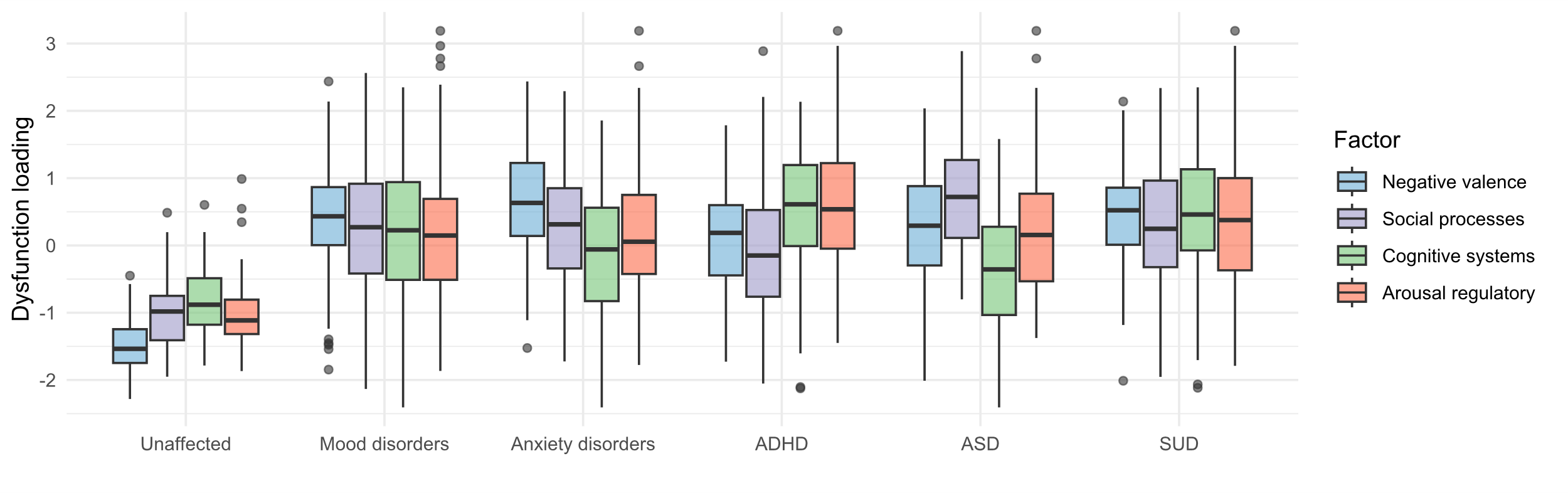
**

**Figure S5. Factor loadings per diagnostic group.** The median per factor per group is shown in each box plot; the lower and upper hinges correspond to the 1^st^ and 3^rd^ quartiles; the whiskers extend from the hinge to the largest/smallest values no further than 1.5 times the inter-quartile range. Outliers are plotted individually.

**References**

1. Rush, A. J., Gullion, C. M., Basco, M. R., Jarrett, R. B., & Trivedi, M. H. (1996). The Inventory of Depressive Symptomatology (IDS): Psychometric properties. *Psychological Medicine*, *26*(3), 477–486. <https://doi.org/10.1017/S0033291700035558>
2. Reiss, S., Peterson, R. A., Gursky, D. M., & McNally, R. J. (1986). Anxiety sensitivity, anxiety frequency and the prediction of fearfulness. *Behaviour Research and Therapy*, *24*(1), 1–8. <https://doi.org/10.1016/0005-7967(86)90143-9>
3. Conners, C. K., Erhardt, D., & Sparrow, E. P. (1999). *Conners' adult ADHD rating scales (CAARS): technical manual*. North Tonawanda, NY: Multi-Health Systems.
4. Baron-Cohen, S., Wheelwright, S., Skinner, R., Martin, J., & Clubley, E. (2001). The Autism-Spectrum Quotient (AQ): Evidence from Asperger Syndrome/High-Functioning Autism, Malesand Females, Scientists and Mathematicians. *Journal of Autism and Developmental Disorders*, *31*(1), 5–17. <https://doi.org/10.1023/A:1005653411471>
5. Díaz-Batanero, C., Ramírez-López, J., Domínguez-Salas, S., Fernández-Calderón, F., & Lozano, Ó. M. (2019). Personality Inventory for DSM-5–Short Form (PID-5-SF): Reliability, Factorial Structure, and Relationship With Functional Impairment in Dual Diagnosis Patients. *Assessment*, *26*(5), 853–866. <https://doi.org/10.1177/1073191117739980>
6. Leising, D., Grande, T., & Faber, R. (2009). The Toronto Alexithymia Scale (TAS-20): A measure of general psychological distress. *Journal of Research in Personality*, *43*(4), 707–710. <https://doi.org/10.1016/j.jrp.2009.03.009>
7. Roth, R. M., & Gioia, G. A. (2005). *Behavior rating inventory of executive function--adult version*. Lutz, FL: Psychological Assessment Resources.
8. Ehring, T., Zetsche, U., Weidacker, K., Wahl, K., Schönfeld, S., & Ehlers, A. (2011). The Perseverative Thinking Questionnaire (PTQ): Validation of a content-independent measure of repetitive negative thinking. *Journal of Behavior Therapy and Experimental Psychiatry*, *42*(2), 225–232. <https://doi.org/10.1016/j.jbtep.2010.12.003>
9. Purves, K. L., Coleman, J. R. I., Meier, S. M., Rayner, C., Davis, K. A. S., Cheesman, R., Bækvad-Hansen, M., Børglum, A. D., Wan Cho, S., Jürgen Deckert, J., Gaspar, H. A., Bybjerg-Grauholm, J., Hettema, J. M., Hotopf, M., Hougaard, D., Hübel, C., Kan, C., McIntosh, A. M., Mors, O., … Eley, T. C. (2020). A major role for common genetic variation in anxiety disorders. *Molecular Psychiatry*, *25*(12), 3292–3303. <https://doi.org/10.1038/s41380-019-0559-1>
10. Demontis, D., Walters, G. B., Athanasiadis, G., Walters, R., Therrien, K., Nielsen, T. T., Farajzadeh, L., Voloudakis, G., Bendl, J., Zeng, B., Zhang, W., Grove, J., Als, T. D., Duan, J., Satterstrom, F. K., Bybjerg-Grauholm, J., Bækved-Hansen, M., Gudmundsson, O. O., Magnusson, S. H., … Børglum, A. D. (2023). Genome-wide analyses of ADHD identify 27 risk loci, refine the genetic architecture and implicate several cognitive domains. *Nature Genetics*, *55*(2), Article 2. <https://doi.org/10.1038/s41588-022-01285-8>
11. Grove, J., Ripke, S., Als, T. D., Mattheisen, M., Walters, R. K., Won, H., Pallesen, J., Agerbo, E., Andreassen, O. A., Anney, R., Awashti, S., Belliveau, R., Bettella, F., Buxbaum, J. D., Bybjerg-Grauholm, J., Bækvad-Hansen, M., Cerrato, F., Chambert, K., Christensen, J. H., … Børglum, A. D. (2019). Identification of common genetic risk variants for autism spectrum disorder. *Nature Genetics*, *51*(3), 431–444. <https://doi.org/10.1038/s41588-019-0344-8>
12. Mullins, N., Forstner, A. J., O’Connell, K. S., Coombes, B., Coleman, J. R. I., Qiao, Z., Als, T. D., Bigdeli, T. B., Børte, S., Bryois, J., Charney, A. W., Drange, O. K., Gandal, M. J., Hagenaars, S. P., Ikeda, M., Kamitaki, N., Kim, M., Krebs, K., Panagiotaropoulou, G., … Andreassen, O. A. (2021). Genome-wide association study of more than 40,000 bipolar disorder cases provides new insights into the underlying biology. *Nature Genetics*, *53*(6), 817–829. <https://doi.org/10.1038/s41588-021-00857-4>
13. Trubetskoy, V., Pardiñas, A. F., Qi, T., Panagiotaropoulou, G., Awasthi, S., Bigdeli, T. B., ... & Lazzeroni, L. C. (2022). Mapping genomic loci implicates genes and synaptic biology in schizophrenia. *Nature, 604*(7906), 502-508. <https://doi.org/10.1038/s41586-022-04434-5>
14. Wray, N. R., Ripke, S., Mattheisen, M., Trzaskowski, M., Byrne, E. M., Abdellaoui, A., Adams, M. J., Agerbo, E., Air, T. M., Andlauer, T. M. F., Bacanu, S.-A., Bækvad-Hansen, M., Beekman, A. F. T., Bigdeli, T. B., Binder, E. B., Blackwood, D. R. H., Bryois, J., Buttenschøn, H. N., Bybjerg-Grauholm, J., … Sullivan, P. F. (2018). Genome-wide association analyses identify 44 risk variants and refine the genetic architecture of major depression. *Nature Genetics*, *50*(5), 668–681. <https://doi.org/10.1038/s41588-018-0090-3>
15. Howard, D. M., Adams, M. J., Clarke, T.-K., Hafferty, J. D., Gibson, J., Shirali, M., Coleman, J. R. I., Hagenaars, S. P., Ward, J., Wigmore, E. M., Alloza, C., Shen, X., Barbu, M. C., Xu, E. Y., Whalley, H. C., Marioni, R. E., Porteous, D. J., Davies, G., Deary, I. J., … McIntosh, A. M. (2019). Genome-wide meta-analysis of depression identifies 102 independent variants and highlights the importance of the prefrontal brain regions. *Nature Neuroscience*, *22*(3), 343–352. <https://doi.org/10.1038/s41593-018-0326-7>
16. Howard, D. M., Adams, M. J., Shirali, M., Clarke, T. K., Marioni, R. E., Davies, G., ... & McIntosh, A. M. (2018). Genome-wide association study of depression phenotypes in UK Biobank identifies variants in excitatory synaptic pathways. *Nature communications*, *9*(1), 1-10. <https://doi.org/10.1038/s41467-018-03819-3>
17. Lee, P. H., Anttila, V., Won, H., Feng, Y.-C. A., Rosenthal, J., Zhu, Z., Tucker-Drob, E. M., Nivard, M. G., Grotzinger, A. D., Posthuma, D., Wang, M. M.-J., Yu, D., Stahl, E. A., Walters, R. K., Anney, R. J. L., Duncan, L. E., Ge, T., Adolfsson, R., Banaschewski, T., … Smoller, J. W. (2019). Genomic Relationships, Novel Loci, and Pleiotropic Mechanisms across Eight Psychiatric Disorders. *Cell*, *179*(7), 1469-1482.e11. <https://doi.org/10.1016/j.cell.2019.11.020>
18. Horn, J. L. (1965). A rationale and test for the number of factors in factor analysis. *Psychometrika, 30*(2), 179–185. <https://doi.org/10.1007/BF02289447>
19. Patil Vivek H, Surendra N. Singh, Sanjay Mishra, and D. Todd Donavan (2017). Parallel Analysis Engine to Aid in Determining Number of Factors to Retain using R [Computer software], available from <https://analytics.gonzaga.edu/parallelengine/>.
